# COVID-19 Incidence and Age Eligibility for Elementary School

**DOI:** 10.1001/jamanetworkopen.2024.44836

**Published:** 2024-11-14

**Authors:** Eve Lin, Alyssa Bilinski, Philip A. Collender, Vivian Lee, Sohil R. Sud, Tomás M. León, Lauren A. White, Justin V. Remais, Jennifer R. Head

**Affiliations:** 1 College of Letters and Sciences, University of California, Berkeley; 2 School of Public Health, Brown University, Providence, Rhode Island; 3 Division of Environmental Health Sciences, University of California, Berkeley; 4 California Department of Public Health, Richmond; 5 Department of Epidemiology, University of Michigan, Ann Arbor; 6 Institute of Global Change Biology, University of Michigan, Ann Arbor

## Abstract

**Importance:**

Understanding the role of school attendance on transmission of SARS-CoV-2 among children is of importance for responding to future epidemics. Estimating discontinuities in outcomes by age of eligibility for school attendance has been used to examine associations between school attendance and a variety of outcomes, but has yet to be applied to describe associations between school attendance and communicable disease transmission.

**Objective:**

To estimate the association between eligibility for elementary school and COVID-19 incidence.

**Design, Setting, and Participants:**

This case series used data on all pediatric COVID-19 cases reported to California’s disease surveillance system between May 16, 2020, and December 15, 2022, among children within 24 months of the age threshold for school eligibility.

**Exposure:**

Birthdate before or after the age threshold for elementary school eligibility during periods when school was remote vs in person.

**Main Outcomes and Measures:**

COVID-19 cases and hospitalizations.

**Results:**

Between May 16, 2020, and December 15, 2022, there were 688 278 cases of COVID-19 (348 957 cases [50.7%] among boys) and 1423 hospitalizations among children who turned 5 years within 24 months of September 1 of the school year when their infection occurred. The mean (SD) age of the study sample was 5.0 (1.3) years. After adjusting for higher rates of testing in schooled populations, the estimated pooled incidence rate ratio among kindergarten-eligible individuals (eg, those born just before the age threshold for school eligibility) compared with those born just after the eligibility threshold for in-person fall 2021 semester was 1.52 (95% CI, 1.36-1.68), for in-person spring 2022 semester was 1.26 (95% CI, 1.15-1.39), and for in-person fall 2022 semester was 1.19 (95% CI, 1.03-1.38). Reported incidence rates among school-eligible children remained higher during the month-long winter 2021-2022 school break but were lower during the longer summer break that followed. The findings were unable to establish whether associations between school eligibility and COVID-19 incidence were based on in-school vs out-of-school routes (eg, classrooms vs school buses). The study lacked power to detect associations between school attendance and hospitalization. Results were robust to functional form. A simulation study was conducted to demonstrate bias associated with nonadjustment for differential case acquisition by exposure status.

**Conclusions and Relevance:**

In this case series of children in California, the magnitude of the association between school eligibility and COVID-19 incidence decreased over time and was generally lower than other published associations between out-of-school child social interactions and COVID-19 incidence. This regression discontinuity design approach could be adapted to other geographies and/or disease systems to assess associations between schooling and disease transmission.

## Key Points

**Question:**

Was eligibility for elementary school associated with COVID-19 infection in California?

**Finding:**

In this case series study, using regression discontinuity methods that adjusted for differential testing rates in schooled populations, higher incidences of COVID-19 were found among California children eligible for kindergarten compared with children born just after the age threshold for school eligibility during in-person semesters: fall 2021 (51.5% higher), spring 2021 (26.3% higher), and fall 2022 (19.1% higher). No associations were found between school eligibility and hospitalization.

**Meaning:**

This study suggests that associations between school eligibility and COVID-19 incidence decreased over time and were generally smaller than published associations between out-of-school gatherings and incidence.

## Teaser

This case series used data on pediatric COVID-19 cases reported to California’s disease surveillance system to estimate the association between eligibility for elementary school and COVID-19 incidence.

## Introduction

Although the association of school attendance with the transmission of SARS-CoV-2 among children emerged as one of the most important questions of the pandemic, it proved difficult to answer.^1^ Ecological studies examining changes in case growth rates or reproduction number before and after school closures were limited in their ability to disentangle the effect of school interventions from concurrent policies (eg, workplace closures, social gathering bans).^2,3^ Prospective studies observing students in classroom settings provided some evidence that COVID-19 incidence among students mimicked rates in the general community, while offering methodological benefits such as controlled testing and examination of multiple end points.^4,5,6,7,8^ However, these studies can be costly to conduct and are too time consuming during early stages of a pandemic, when decisions must be made quickly. Furthermore, research suggests that the effect of schooling on COVID-19 incidence depends on local mitigation efforts^1^ and vaccination rates,^9^ limiting generalizability of findings from focal cohort studies.

Given the substantial tolls of school closures on childhood education^10,11^ and mental health,^12,13^ as well as the exacerbation of social and racial and ethnic inequalities in these outcomes,^10,14,15^ methods are needed to rapidly and robustly quantify the association of school attendance with transmission rates of newly emerged pathogens or variants, including SARS-CoV-2 variants or novel influenza strains. The age threshold for school eligibility (typically whether a child’s fifth birthday falls before September 1)^16^ is a promising instrumental variable for school exposures that can be used within a regression discontinuity design (RDD) to yield insights into outcomes associated with schooling. Because children born immediately before the age threshold for school eligibility are assumed to be similar to those born immediately after, any discontinuous changes in outcomes as age crosses the threshold can be interpreted as being associated, at least in part, with school attendance.^17,18^ Use of age-based school eligibility data avoids the extensive data collection required by cohort studies or the experimentation in policy necessary for ecological studies.

**Figure 1.**
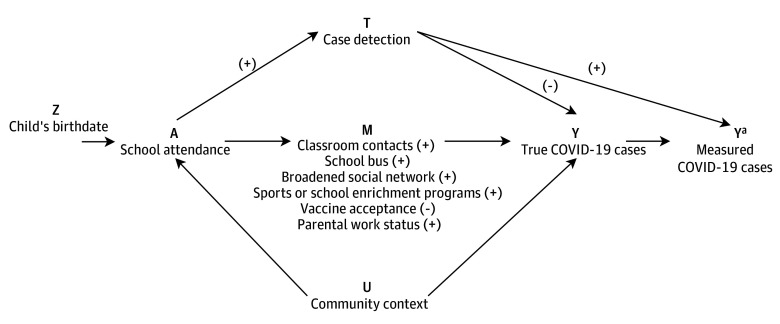
Directed Acyclic Graph Representing Associations Between Child Birthdate, School Attendance, and COVID-19 Cases (+) Indicates a positive association; (−) indicates a negative association. ^a^Signifies the measured version vs the true version.

Regression discontinuity designs have been used to examine associations between school attendance and crime initiation,^19^ adult earnings,^20^ adolescent obesity,^21^ and maternal anxiety,^22^ but, to our knowledge, have not been applied to understand the association between school attendance and infectious disease outcomes.^23^ School attendance may be associated with disease transmission via both in-school contacts and out-of-school consequences of schooling (eg, riding buses, parents returning to work; Figure 1). Although routine collection of data on reportable diseases enhances the feasibility of using RDDs to study associations between school attendance and infectious diseases outcomes, measurement error of the outcome via imperfect case ascertainment may bias effect estimates if case ascertainment is differential by exposure status. This concern is salient for COVID-19, as in-school symptomatic and asymptomatic testing programs were a component of many COVID-19 prevention plans (Figure 1).

Here, we explore RDD as a means of estimating the association between school attendance and an infectious disease in the presence of differential case ascertainment by comparing COVID-19 incidence and hospitalizations among children born just after the age threshold for kindergarten eligibility with those born just before. California was chosen as the setting for this study because it has the largest public school enrollment in the US^24^ and is the most socioeconomically diverse state.^25^

## Methods

### Data Source

We obtained information on all pediatric COVID-19 cases reported in California between May 16, 2020, and December 15, 2022, from the California COVID-19 Reporting System. COVID-19 cases were confirmed using a positive nucleic acid amplification test. Each record contained information on the patient’s census tract of residence, age in months, and hospitalization status. Because the underlying data were collected for public health surveillance and deidentified, the secondary research described herein is exempt from institutional review board review and consent according to the Common Rule (45 CFR §46). We followed the reporting guidelines for case series studies.

We defined school periods as fall semester (August 15-December 14), winter break (December 15-January 14), spring semester (January 15-May 15), and summer break (May 16-August 14) (eFigure 5 and eTable 3 in Supplement 1).^26,27^ We examined 2 semesters of remote instruction (2020-2021 academic year) and 3 semesters of in-person instruction (fall and spring of the 2021-2022 academic year and fall of the 2022-2023 academic year).

For each academic year being analyzed, we assessed the subsample of individuals within the surveillance record who would fall within a given bandwidth, *h*, of the age threshold for school eligibility (eTable 3 in Supplement 1). In California, as in most states, children must be 5 years of age by September 1 to enter kindergarten. Within each county and school period (ie, fall and spring semesters, summer and winter breaks), we calculated the number of reported cases, stratified by child’s birth month. Hospitalization data were summarized similarly to case data, except we summed cases to the state level, rather than county level, due to the relative rarity of hospitalizations.

To obtain population denominators, we used data on births per county and month that gave rise to the underlying population.^28^ For instance, for the 2020-2021 academic year, we obtained county-level data on the number of children born each month in the bandwidth around September 1, 2015 (eTable 3 in Supplement 1).

### Statistical Analysis

#### Regression Discontinuity Design

We used a sharp RDD to estimate the association between school attendance and reported COVID-19 cases and hospitalizations among young children, assuming that age at September 1 is associated with whether or not an individual attends school that year.

For the sample of data with –*h* *<* *x_i_<* *h*, where *h* is bandwidth and *x_i_* is the difference in age (in months) from the school eligibility threshold on September 1 of the relevant period, grouped into 1-month bins, we ran Poisson regressions with the form: log [*E*(*Y_i_*,|*Z_i_x_i_*)]= log(*b_i_*) + α + τ*Z_i_*>+ *f*(*x_i_*) + *g*(*x_i_Z_i_*), where *Y_i_* is the number of COVID-19 cases or hospitalizations among children in age bin *i*; *b_i_* is the number of births of children in age bin *i*, approximating population size; *Z_i_* is a binary indicator for school eligibility in the current period (ie, whether, *x_i_* *>* *0*); *f* is a function associating age with the outcome among noneligible children; *g* is a function describing the modification of the association between age and outcome among eligible children; α is an intercept term; and τ, the target parameter, is the coefficient describing the marginal association of eligibility for school attendance with the outcome. Exponentiating τ yields the incidence rate ratio (IRR) comparing COVID-19 among children born just before the age threshold for school eligibility with children born just after the threshold. Because our primary exposure is age eligibility for school attendance rather than attendance, the estimated IRR is considered an intention-to-treat effect estimate.^29^ We used 95% CIs to indicate statistical significance.

To contend with bias from differential case ascertainment, we weighted cases in the school-ineligible strata to approximate the number of cases that would have been observed in the school-ineligible age groups given equal testing effort (see next section). We did not apply weights for analyses with hospitalization as the outcome, as ascertainment of severe cases is more likely to be similar between populations.

Children born between September 2 and December 2 may have attended California’s transitional kindergarten (TK) program.^30^ Although TK classes are smaller than kindergarten classes, TK may result in similar exposures as elementary school attendance. In main analyses, we removed individuals eligible for TK and we included them in sensitivity analyses.

For cases, we fit separate models for 46 of California’s 58 counties, excluding 12 counties with few cases and births reported. We pooled IRRs using a meta-analysis approach that has been used to combine effect estimates across multiple locations while examining effect heterogeneity (R mvmeta package; eAppendix in Supplement 1).^31^ All analyses were conducted in R, version 4.2.0 (R Project for Statistical Computing).^32^

#### Adjustment for Differential Case Ascertainment

As case detection was a feature of California’s reopening plans for kindergarten through grade 12, testing rates were higher among school-aged children compared with younger children (eFigure 6 in Supplement 1). To contend with bias from differential case ascertainment, we conducted a simulation study to identify a weighting factor, that, when used to upweight cases in the school-ineligible strata, generated an estimated IRR that most closely approximated the true IRR (eAppendix in Supplement 1). Briefly, we used a susceptible–exposed–infectious (asymptomatic/symptomatic)–recovered model to simulate both true and observed cases according to the hypothesized data-generating mechanism and expected transmission parameters for the study population (eFigures 1 and 2 and eTables 1 and 2 in Supplement 1). We then fit a regression discontinuity model to the simulated true and observed cases to calculate true and observed IRRs. Upweighting cases in the school-ineligible strata by the square root of the county’s testing ratio for the 5- to 10-year age group vs the 0- to 4-year age group best allowed the observed IRR to approximate the true IRR (eAppendix and eFigures 3 and 4 in Supplement 1), a result consistent with adjustment functions arrived at in other studies using alternative means of estimation.^33^ To provide another bound on estimated IRRs, we upweighted cases in the school-ineligible strata by the county’s testing ratio for the 5- to 10-year age group vs the 0- to 4-year age group. This approach is considered conservative because testing was conducted as a screening tool among asymptomatic populations, so each test administered had a lower probability of a positive result.

#### Sensitivity Analyses, Negative Controls, and Power Simulation

To assess the robustness of results to model specification, we ran analyses varying bandwidth *h* from 8 to 24 months and testing 3 different functional bases (linear, quadratic, locally linear, or locally estimated scatterplot smoothing [loess]) for the association of age *x_i_* with COVID-19 outcomes (eAppendix in Supplement 1). We compared model fit using the Akaike information criterion (AIC).^34,35^

As a negative control to identify the presence of unmeasured confounders, we examined whether there were discontinuities in COVID-19 incidence at the age-eligibility threshold during periods when we should not expect an increase in cases, including semesters when school was remote and summer and winter breaks prior to reopening of in-person instruction.

Insufficient power can be a limitation of regression discontinuity analyses,^36^ a concern salient for our hospitalization analysis given the relative rarity of the outcome. We performed a power simulation study to examine the plausibility that associations of school eligibility with hospitalization were not detected due to rarity of the hospitalization outcome (eAppendix in Supplement 1).^37^

## Results

### Population Characteristics

Between May 16, 2020, and December 15, 2022, there were 688 278 cases of COVID-19 (348 957 [50.7%] among boys and 339 321 [49.3%] among girls) and 1423 hospitalizations among children who turned 5 years within 24 months of September 1 of the school year when their infection occurred. The mean (SD) age of the study sample was 5.0 (1.3) years. The number of cases reported during in-person semesters ranged from 28 088 to 212 093 cases and 121 to 318 hospitalizations (eTable 3 in Supplement 1).

### Association Between Elementary School Attendance and Reported COVID-19 Cases

For 36 (78.3%) of the 46 counties examined, models with linear associations between age and COVID-19 incidence had lower AICs than models using local linear regression or quadratic terms. Here, we present results from linear models fitted using a bandwidth *h* of 24 months.

**Figure 2.**
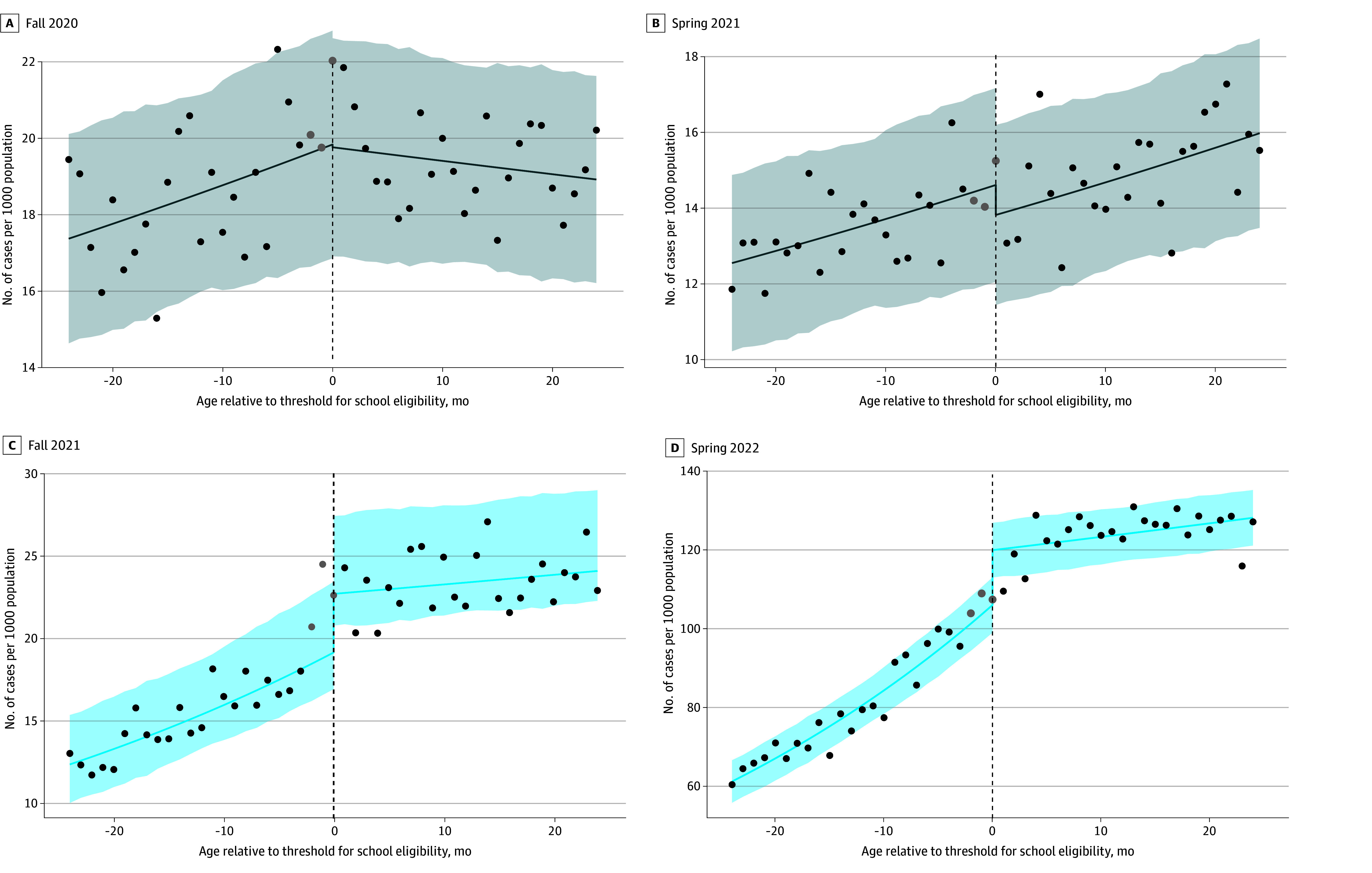
COVID-19 Incidence as a Function of Children’s Age Relative to the September 1 Age Threshold for School Eligibility Ages to the right of the threshold indicate that the child is age eligible to attend elementary school (kindergarten through grade 5). Model fits are shown for the fall 2020 (A) and spring 2021 (B) semesters when school was remote and during the fall 2021 (C) and spring 2022 (D) semesters when school was in-person. Dots indicate observed data, the gray dots indicate data for the children who were eligible for transitional kindergarten and excluded from main analyses, the lines indicate model fit, and the shaded regions indicate 95% CIs. For this example, weighting to adjust for testing biases is not performed. Plots shown are selected from Los Angeles County, which contains the largest school district in California. Models shown assume a linear association between age and incidence, use a bandwidth of 24 months, and exclude children eligible for transitional kindergarten. Fits from other functional forms are shown in eFigures 7 and 8 in Supplement 1.

**Figure 3.**
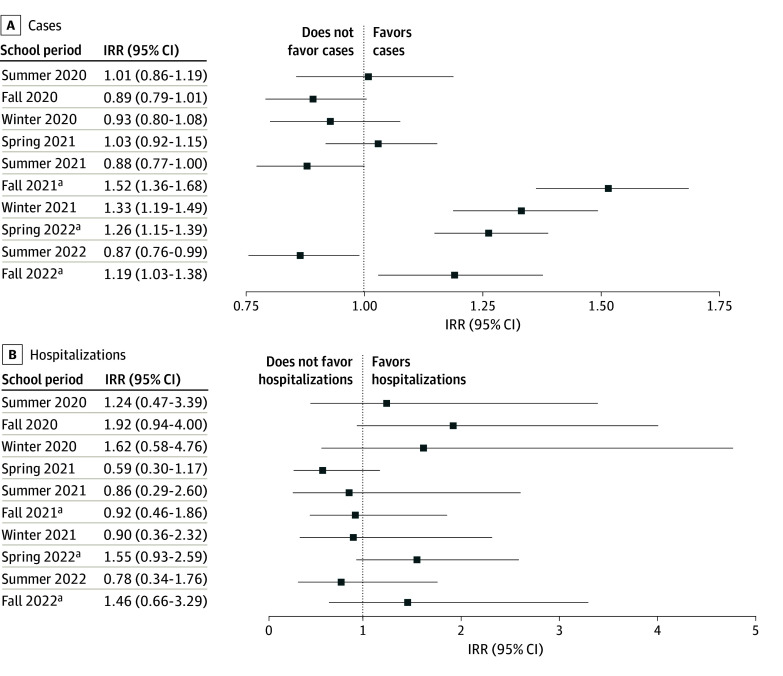
Incidence of COVID-19 Cases and Hospitalizations Incidence rate ratios (IRRs) and 95% CIs (indicated by horizontal lines) representing incidence of COVID-19 cases (pooled across counties) (A) and hospitalizations (statewide) (B) among children born just before the threshold for elementary school attendance (kindergarten through grade 5) compared with those born just after. Models shown assume a linear association between age and outcome, use a bandwidth of 24 months, exclude children eligible for transitional kindergarten (TK), and adjust for bias due to unequal case ascertainment. eFigure 13 in Supplement 1 shows results including individuals eligible for TK. ^a^Periods when schools were open for in-person instruction.

Reported COVID-19 incidence was higher among children eligible for school attendance during in-person semesters in the study period (Table; Figure 2 shows unweighted visual discontinuities for Los Angeles County; eFigures 7 and 8 in Supplement 1 show visual discontinuities using different functional forms). Using our best correction for differential case ascertainment, the pooled estimate of the school eligibility IRR for COVID-19 during the fall 2021 semester was 1.52 (95% CI, 1.36-1.68) (Table and Figure 3A), meaning that age-adjusted incidence rates were 51.5% (95% CI, 36.3%-68.5%) higher among children eligible for elementary school (eg, those born just before the age threshold for school eligibility) compared with ineligible children. The IRR for the winter break that followed the fall 2021 semester was 1.33 (95% CI, 1.19-1.49). Incidence rate ratios for the spring 2022 semester were 1.26 (95% CI, 1.15-1.39) and the fall 2022 semester was 1.19 (95% CI, 1.03-1.38). Age-adjusted incidence rates for the spring 2022 semester were 26.3% (95% CI, 14.9%-38.8%) higher and for the fall 2022 semester were 19.1% (95% CI, 3.0%-37.3%) higher among children eligible for elementary school compared with ineligible children. eFigures 9 to 11 in Supplement 1 show county-specific IRRs.

**Table.** Incidence Rate Ratios Comparing the Incidence of COVID-19 Among Children Born Just Before the Threshold for Elementary School Attendance (September 1) Compared With Just After

School period	Incidence rate ratio (95% CI)	
	Excluding TK-eligible children^a^	Including TK-eligible children^a^
Prior to reopening of schools for in-person instruction		
Summer 2020	1.01 (0.86-1.19)	1.00 (0.96-1.16)
Fall 2020	0.89 (0.79-1.01)	0.95 (0.85-1.06)
Winter 2020	0.93 (0.80-1.08)	0.96 (0.84-1.10)
Spring 2021	1.03 (0.92-1.15)	1.03 (0.93-1.14)
Summer 2021	0.88 (0.77-1.00)	0.94 (0.84-1.06)
After reopening of schools for in-person instruction		
Fall 2021^b^	1.52 (1.36-1.68)	1.23 (1.13-1.34)
Winter 2021	1.33 (1.19-1.49)	1.24 (1.12-1.37)
Spring 2022^b^	1.26 (1.15-1.39)	1.11 (1.02-1.19)
Summer 2022	0.87 (0.76-0.99)	0.91 (0.80-1.03)
Fall 2022^b^	1.19 (1.03-1.38)	1.06 (0.94-1.21)

Abbreviation: TK, transitional kindergarten.

^a^ Children born between September 2 and December 1 are eligible for TK, so we conducted analyses both excluding and including children born from September 2 to December 1.

^b^ In-person semester included in the study period.

Including individuals eligible for TK resulted in effect estimates closer to the null (Table; eFigures 12 and 13 in Supplement 1). Using the more conservative weighting factor resulted in effect estimates closer to the null, which were significant only for the 2021-2022 academic year (fall 2021 IRR, 1.36 [95% CI, 1.20-1.53]; spring 2022 IRR, 1.14 [95% CI, 1.03-1.28]) (eTable 4 and eFigure 12 in Supplement 1). Not adjusting for differential testing resulted in effect estimates further from the null. This finding aligns with results from our simulation study, which demonstrates deviation between the true and observed IRR when outcome measurement is associated with exposure (eAppendix in Supplement 1).

**Figure 4.**
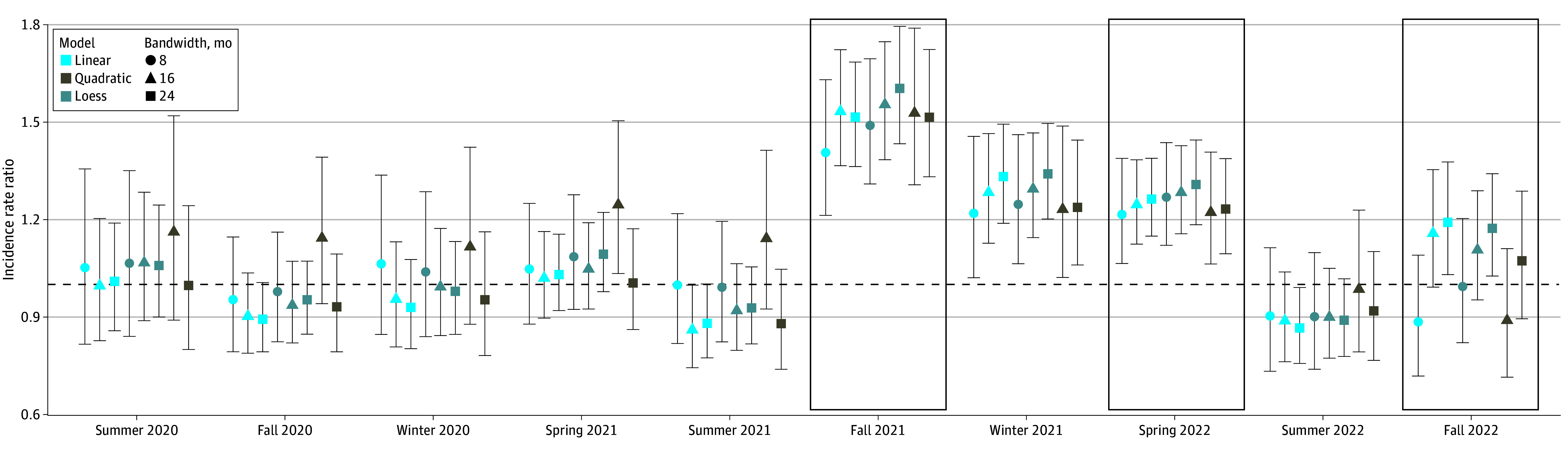
Incidence of COVID-19 Cases and Hospitalizations Incidence rate ratios and 95% CIs (indicated by vertical lines) representing incidence of COVID-19 cases among children born just before the threshold for elementary school attendance (kindergarten through grade 5) compared with those born just after. Incidence rate ratios are displayed for various model parametrizations (eg, local linear regression [locally estimated scatterplot smoothing (Loess)], linear association between age and incidence, and quadratic association between age and incidence) and bandwidths. In-person semesters are outlined in black boxes. Quadratic models with 8-month bandwidths are not shown due to large CIs. Graphical results for hospitalizations are displayed in eFigure 18 in Supplement 1.

No significant differences in COVID-19 case incidence were observed during academic periods prior to the reopening of schools for in-person instruction (Table; Figure 3A). Reported incidence remained higher among school-eligible children during the month-long winter 2021-2022 break that followed the in-person fall 2021 semester (IRR, 1.33 [95% CI, 1.19-1.49]), and was lower during the longer summer 2022 break that followed in-person 2021-2022 academic year (IRR, 0.87 [95% CI, 0.76-0.99]). Results were robust to functional forms (Figure 4).

Of county-level variables examined, only population explained heterogeneity in school eligibility IRR across counties in meta-regression (eTable 5 in Supplement 1). Higher county population was associated with lower IRRs across all 3 in-person semesters, suggesting that associations between schooling and COVID-19 incidence were weaker among more populous counties. Counties with a higher proportion of the population who reported never wearing a mask and with a higher percentage of single-parent families had higher school eligibility IRRs across all 3 in-person semesters, but this association was not significant at the 95% confidence level.

### Association Between Elementary School Attendance and Reported COVID-19 Hospitalizations

There were no discernible increases in reported age-adjusted COVID-19 hospitalizations rates among children who were born before the school attendance age threshold for any period examined (eFigures 14-16 in Supplement 1) and alternative model parameterizations (eFigure 17 in Supplement 1). This finding could have been due to the rarity of hospitalization for COVID-19 among children (eFigure 18 and eAppendix in Supplement 1).

## Discussion

Using a regression discontinuity approach and adjusting for greater case ascertainment among school-aged populations, we estimated increases in COVID-19 incidence of 51.5% during the fall 2021 semester, 26.3% during the spring 2022 semester, and 19.1% during the fall 2022 semester among children born just before the age threshold for kindergarten eligibility in California compared with children born just after during the first 3 semesters when schools were open for in-person instruction. Overall, the estimated effect sizes for the 3 in-person semesters are similar in magnitude to or smaller than the estimated effect size of child social gatherings (increase of 31% in COVID-19 incidence after birthdays)^38^ and smaller than the effect size associated with large gatherings among adults.^39^

Our findings on associations between in-person school attendance and COVID-19 case incidence align with prior research. Although reported outbreaks among classroom and daycare settings suggest that these locations hold potential for spreading disease,^40,41,42,43,44^ evidence suggests that precautions were able to effectively mitigate large increases in transmission.^4,5,11^ Prior work also demonstrates slight increases in incidence among teachers^45^ and household members of students, which can be minimized by in-school prevention measures.^1^ Model-based^9,46^ and empirical studies have found limited associations between schooling and severe pediatric COVID-19,^11,47,48^ which, as in our study, may be associated with the relative rarity of severe outcomes among children. We found that COVID-19 incidence increases linearly with age, controlling for school eligibility (Figure 2), a trend that has been observed elsewhere.^49^ When considering the evidence associating school closures with learning loss,^10^ adverse mental health,^12,13^ and widening disparities,^14,15^ our findings and those of others support the use of within-school cautionary measures as much as possible over school closures.^50^

The association between school eligibility and COVID-19 incidence decreased with each subsequent in-person semester. In most model parameterizations, school eligibility appeared to be associated with protection against COVID-19 incidence during the summer after the first in-person school year that followed widespread school closures. These findings may suggest a protective association of natural immunity due to higher infection rates and/or greater adoption of vaccines, which became available in late October of the fall 2021 semester.^51^

In meta-analyses, we found that more populated counties had weaker associations between in-person schooling and COVID-19 incidence. This finding could reflect the fact that major school districts in populated counties, including Los Angeles, San Diego, San Francisco, and Sacramento, adhered to stricter within-school mitigation measures, including longer mask mandates.^52^ The percentage of the population who reported never wearing a mask was associated with stronger associations between in-person schooling and COVID-19 incidence, although not significantly so at the 95% confidence level. More populous counties also had a higher cumulative incidence of COVID-19 at the time of school reopening, suggesting higher immunity of schooled children and/or increased transmission among the comparison group of school-ineligible children.

### Limitations

This study has some limitations. We cannot identify whether higher COVID-19 incidence among children born before vs after the school attendance threshold are due to in-school or out-of-school exposures that are associated with school attendance, such as bus ridership or sports programs (Figure 1). Prior work suggests that such community settings pose a higher risk than classrooms of infection.^53^ Second, the comparison group in this study—children younger than the threshold for school attendance—has heterogeneous contact patterns that could vary between staying at home or being placed in daycare. We treated our RDD as a sharp design, assuming all children eligible for school will attend school, and all children not eligible for school will not attend school. However, children may be homeschooled, held back, or sent to school early. The resulting bias from exposure misclassification is expected to be toward the null.

We modeled surveillance data, so jumps in discontinuities at the threshold may reflect increased testing, a bias we attempted to adjust for in analyses. The estimated IRR for the fall 2021 semester (1.52) is similar to that for the winter break that followed (1.33), providing some validity for our bias-adjusted estimate, as school-associated disparities in testing volume should have been smaller over the winter break period, yet elevated incidence associated with school attendance could have lingered due to latency periods and secondary transmission. The testing data we used to estimate adjustment factors did not account for differential testing over time, which would have permitted use of alternative methods that have been published that compare incidences adjusted for differential testing.^54^ Nevertheless, our weighting factor is consistent with previous literature that uses convex functions of testing effort to adjust ascertained cases upward.^33,54^ Here, we estimate function to be a square root function, which is consistent with that estimated by other study.^33^ The true IRRs may thus be closer to the null if the full extent of disparities in testing volume were dampened by inclusion of periods when school was on break. In-school testing efforts may have tapered over time, contributing, in part, to the observed decrease in IRRs over time. The challenge of differential case ascertainment by school status is likely greater for COVID-19 than for other pediatric infections, such as influenza or respiratory syncytial virus, where in-school surveillance testing is not common.

The effect estimates from this study may not be generalizable to states outside California, which held longer masking policies than most states and had vaccine mandates for teachers,^55^ nor other countries that may have kept schools open while maintaining more stringent contact tracing or other within-school measures. Although we could not examine associations between school eligibility and COVID-19 incidence among potentially more vulnerable household members of schooled children, this approach could be used to investigate this association, if information on the age of household members of adult cases was known.

## Conclusions

In this case series of pediatric COVID-19 cases, we estimated increases in reported COVID-19 incidence among children eligible for elementary school attendance in California compared with children ineligible for elementary school, and observed that these increases decreased over time. This study demonstrated the feasibility of regression discontinuity to investigate associations between COVID-19 and school attendance. This approach may be especially useful when direct observation and follow-up of school-aged children is not feasible. The novelty of using this approach to answer questions surrounding infectious disease incidence was both a strength of this study and motivator for future research to characterize sources of bias when using the regression discontinuity approach to study infectious disease transmission within schools.

## Notes

Supplement 1.
eAppendix.
**eFigure 1.** Directed Acyclic Graphs (DAGs) Representing Instrumental Variable Analyses With Various Types of Measurement Error **eFigure 2.** SEIR Model Used to Simulate Data According to the Hypothesized Data Generating Mechanism **eTable 1.** Parameter Values Used in the Simulation of Data **eTable 2.** Scenarios From eFigure 1 Were Examined Using the Following Combinations of Parameters **eFigure 3.** Comparison of Observed and True Incidence Rate Ratios (A) and True and Adjusted Incidence Rate Rates (B) **eFigure 4.** Heat Map Comparison of Observed and True Incidence Rate Ratios (A) and True and Adjusted Incidence Rate Rates (B) **eFigure 5.** Timeline of Circulating SARS-CoV-2 Variants, Vaccination Approval Dates, and School Periods **eFigure 6.** Distribution of Testing Rate Ratios and Test Positivity Rate Ratios Among California Counties Comparing Testing Rates and Test Positivity Rates Among School-Aged Children (5-10 Years) to Non-School-Aged Children (0-4 Years) **eFigure 7.** COVID-19 Incidence as a Function of Child Age Relative to the September 1st Cutoff for Elementary School Attendance, for Models Assuming a Quadratic Relationship Between Age and Incidence **eFigure 8.** COVID-19 Incidence as a Function of Child Age Relative to the September 1st Cutoff for Elementary School Attendance, for Models That Use Local Linear Regression **eFigure 9.** County-Specific (Black) and Pooled (Blue) Incidence Rate Ratios (IRR) Representing the Incidence of COVID-19 in the Fall 2021 Semester of the 2021-2022 Academic Year Among Children Born Just Before the Age-Eligibility Threshold for Elementary School Compared to Those Born Just After **eFigure 10.** County-Specific (Black) and Pooled (Blue) Incidence Rate Ratios (IRR) Representing the Incidence of COVID-19 in the Spring 2022 Semester of the 2021-2022 Academic Year Among Children Born Just Before the Age-Eligibility Threshold for Elementary School Compared to Those Born Just After **eFigure 11.** County-Specific (Black) and Pooled (Blue) Incidence Rate Ratios (IRR) Representing the Incidence of COVID-19 in the Fall 2022 Semester of the 2021-2022 Academic Year Among Children Born Just Before the Age-Eligibility Threshold for Elementary School Compared to Those Born Just After **eFigure 12.** Comparison of IRRs During In-School Periods Adjusting for Testing Biases and Not Adjusting for Testing Differences **eFigure 13.** Pooled Incidence Rate Ratios (IRR) Representing the Incidence of COVID-19 Among Children Born Just Before the Age-Eligibility Threshold for Elementary School Compared to Those Born Just After **eFigure 14.** COVID-19 Hospitalization as a Function of Child Age Relative to the September 1st Cutoff for Elementary School Attendance, for Models Assuming a Linear Relationship Between Age and Hospitalizations **eFigure 15.** COVID-19 Hospitalization as a Function of Child Age Relative to the September 1st Cutoff for Elementary School Attendance, for Models Assuming a Quadratic Relationship Between Age and Hospitalizations **eFigure 16.** COVID-19 Hospitalization as a Function of Child Age Relative to the September 1st Cutoff for Elementary School Attendance, for Models That Use Local Linear Regression **eFigure 17.** Associations Between Elementary School Age-Eligibility and Hospitalizations for COVID-19 by School Period and Model Parameterization **eFigure 18.** Power Analysis for the Association Between Hospitalizations and School Eligibility **eTable 3.** Total Number of Cases and Hospitalizations Among the Subsample of Children Who Fell Within 24 Months, in Either Direction, of the Elementary School Attendance Threshold **eTable 4.** Incidence Rate Ratios (IRRs) and 95% Confidence Intervals Comparing the Incidence of COVID-19 Among Children Born Just Before the Threshold for Elementary School Attendance (September 1st) Compared to Just After **eTable 5.** Results of Meta-Analysis
eReferences.

Supplement 2.
Data Sharing Statement
