# Supplementary material for: COVID-19 Incidence and Age Eligibility for Elementary School
Source: JAMA Netw Open. 2024 Nov 14;7(11):e2444836. doi: 10.1001/jamanetworkopen.2024.44836 (PMC12316186; doi:10.1001/jamanetworkopen.2024.44836)
Supplement: Supplement 1. — eAppendix. eFigure 1. Directed Acyclic Graphs (DAGs) Representing Instrumental Variable Analyses With Various Types of Measurement Error eFigure 2. SEIR Model Used to Simulate Data According to the Hypothesized Data Generating Mechanism eTable 1. Parameter Values Used in the Simulation of Data eTable 2. Scenarios From eFigure 1 Were Examined Using the Following Combinations of Parameters eFigure 3. Comparison of Observed and True Incidence Rate Ratios (A) and True and Adjusted Incidence Rate Rates (B) eFigure 4. Heat Map Comparison of Observed and True Incidence Rate Ratios (A) and True and Adjusted Incidence Rate Rates (B) eFigure 5. Timeline of Circulating SARS-CoV-2 Variants, Vaccination Approval Dates, and School Periods eFigure 6. Distribution of Testing Rate Ratios and Test Positivity Rate Ratios Among California Counties Comparing Testing Rates and Test Positivity Rates Among School-Aged Children (5-10 Years) to Non-School-Aged Children (0-4 Years) eFigure 7. COVID-19 Incidence as a Function of Child Age Relative to the September 1st Cutoff for Elementary School Attendance, for Models Assuming a Quadratic Relationship Between Age and Incidence eFigure 8. COVID-19 Incidence as a Function of Child Age Relative to the September 1st Cutoff for Elementary School Attendance, for Models That Use Local Linear Regression eFigure 9. County-Specific (Black) and Pooled (Blue) Incidence Rate Ratios (IRR) Representing the Incidence of COVID-19 in the Fall 2021 Semester of the 2021-2022 Academic Year Among Children Born Just Before the Age-Eligibility Threshold for Elementary School Compared to Those Born Just After eFigure 10. County-Specific (Black) and Pooled (Blue) Incidence Rate Ratios (IRR) Representing the Incidence of COVID-19 in the Spring 2022 Semester of the 2021-2022 Academic Year Among Children Born Just Before the Age-Eligibility Threshold for Elementary School Compared to Those Born Just After eFigure 11. County-Specific (Black) and Pooled (Blue) Incidence Ra [file jamanetwopen-e2444836-s001.pdf]

# Supplementary Online Content

Lin E, Bilinski A, Collender PA, et al. COVID-19 incidence and age eligibility for elementary school.

*JAMA Netw Open.* 2024;7(11):e2444836. doi:10.1001/jamanetworkopen.2024.44836

**eAppendix.**
**eFigure 1.** Directed Acyclic Graphs (DAGs) Representing Instrumental Variable Analyses With Various Types of Measurement Error
**eFigure 2.** SEIR Model Used to Simulate Data According to the Hypothesized Data Generating Mechanism
**eTable 1.** Parameter Values Used in the Simulation of Data
**eTable 2.** Scenarios From eFigure 1 Were Examined Using the Following Combinations of Parameters
**eFigure 3.** Comparison of Observed and True Incidence Rate Ratios (A) and True and Adjusted Incidence Rate Rates (B)
**eFigure 4.** Heat Map Comparison of Observed and True Incidence Rate Ratios (A) and True and Adjusted Incidence Rate Rates (B)
**eFigure 5.** Timeline of Circulating SARS-CoV-2 Variants, Vaccination Approval Dates, and School Periods
**eFigure 6.** Distribution of Testing Rate Ratios and Test Positivity Rate Ratios Among California Counties Comparing Testing Rates and Test Positivity Rates Among School-Aged Children (5-10 Years) to Non-School-Aged Children (0-4 Years)
**eFigure 7.** COVID-19 Incidence as a Function of Child Age Relative to the September 1st Cutoff for Elementary School Attendance, for Models Assuming a Quadratic Relationship Between Age and Incidence
**eFigure 8.** COVID-19 Incidence as a Function of Child Age Relative to the September 1st Cutoff for Elementary School Attendance, for Models That Use Local Linear Regression
**eFigure 9.** County-Specific (Black) and Pooled (Blue) Incidence Rate Ratios (IRR) Representing the Incidence of COVID-19 in the Fall 2021 Semester of the 2021-2022 Academic Year Among Children Born Just Before the Age-Eligibility Threshold for Elementary School Compared to Those Born Just After
**eFigure 10.** County-Specific (Black) and Pooled (Blue) Incidence Rate Ratios (IRR) Representing the Incidence of COVID-19 in the Spring 2022 Semester of the 2021-2022 Academic Year Among Children Born Just Before the Age-Eligibility Threshold for Elementary School Compared to Those Born Just After
**eFigure 11.** County-Specific (Black) and Pooled (Blue) Incidence Rate Ratios (IRR) Representing the Incidence of COVID-19 in the Fall 2022 Semester of the 2021-2022 Academic Year Among Children Born Just Before the Age-Eligibility Threshold for Elementary School Compared to Those Born Just After
**eFigure 12.** Comparison of IRRs During In-School Periods Adjusting for Testing Biases and Not Adjusting for Testing Differences
**eFigure 13.** Pooled Incidence Rate Ratios (IRR) Representing the Incidence of COVID-19 Among Children Born Just Before the Age-Eligibility Threshold for Elementary School Compared to Those Born Just After
**eFigure 14.** COVID-19 Hospitalization as a Function of Child Age Relative to the September 1st Cutoff for Elementary School Attendance, for Models Assuming a Linear Relationship Between Age and Hospitalizations
**eFigure 15.** COVID-19 Hospitalization as a Function of Child Age Relative to the September 1st Cutoff for Elementary School Attendance, for Models Assuming a Quadratic Relationship Between Age and Hospitalizations
**eFigure 16.** COVID-19 Hospitalization as a Function of Child Age Relative to the September 1st Cutoff for Elementary School Attendance, for Models That Use Local Linear Regression
**eFigure 17.** Associations Between Elementary School Age-Eligibility and Hospitalizations for COVID-19 by School Period and Model Parameterization
**eFigure 18.** Power Analysis for the Association Between Hospitalizations and School Eligibility
**eTable 3.** Total Number of Cases and Hospitalizations Among the Subsample of Children Who Fell Within 24 Months, in Either Direction, of the Elementary School Attendance Threshold
**eTable 4.** Incidence Rate Ratios (IRRs) and 95% Confidence Intervals Comparing the Incidence of COVID-19 Among Children Born Just Before the Threshold for Elementary School Attendance (September 1st) Compared to Just After
**eTable 5.** Results of Meta-Analysis
**eReferences.**

This supplementary material has been provided by the authors to give readers additional information about their work.

© 2024 Lin E et al. *JAMA Network Open.*

**eAppendix.**

Simulation study- Methods

This study uses reported COVID-19 cases, which are imperfectly ascertained by reportable disease surveillance systems. We conducted a simulation study in order to understand how measurement error of the outcome biases the estimated incidence rate ratios, and to estimate an adjustment factor that reduces this bias.

First, we drew directed acyclic graphs (DAGs) to visually depict the possible ways in imperfect outcome measurement (here, due to lack of testing) might occur within the study (eFigure 1). These scenarios included: no measurement error; measurement error unassociated with the exposure and the true outcome; measurement error associated with the outcome only or the exposure only; and measurement error associated with both the exposure and the outcome. In this particular case of COVID-19, we believe that measurement error could be associated with both the exposure (higher testing in school aged populations) and the true outcome (case ascertainment leads to case isolation and reduction in cases).

© 2024 Lin E et al. *JAMA Network Open.*

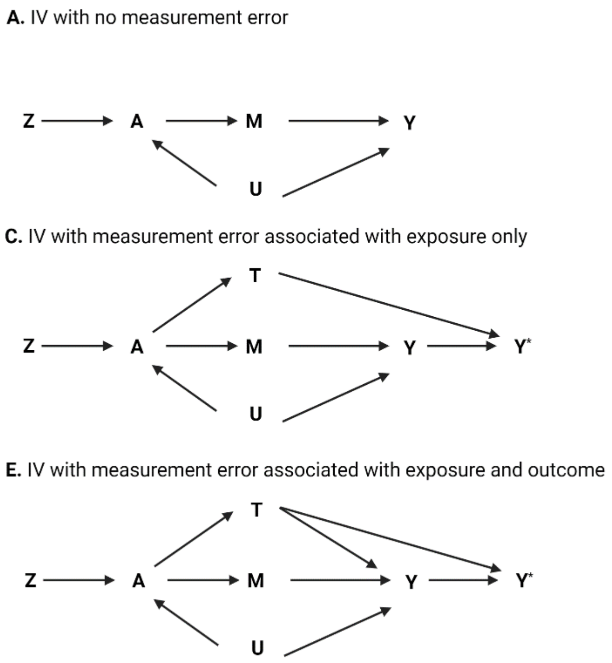


**eFigure 1.** Directed Acyclic Graphs (DAGs) Representing Instrumental Variable Analyses With Various Types of Measurement Error. IV = instrumental variable; Z = running variable (child's birthdate); A = exposure (school attendance); M = mediators (e.g., child social networks inside and outside of classrooms, adult social networks, vaccination willingness, etc.); U = unmeasured confounders (e.g., community controls); Y = true outcome (all COVID cases); Y* = measured outcome (reported COVID cases); T = Testing/case detection.

Next, we simulated data using a compartmental SEIR model (eFigure 2) for each data generating mechanism depicted in eFigure 1 and using the parameter values shown in eTable 1. The SEIR model included compartments for asymptomatic versus symptomatic cases, and tested (i.e., reported) cases versus not tested cases (i.e., not observed). Movement from the susceptible to the exposed compartment was proportional to the force of infection, which was defined as being conditional on age ($a$; via pathogen transmission rate ($\beta$) and probability case is clinical ($p_{C,a}$)) and school attendance ($S$; via contact rates, $K_s$, and case detection, $p_{TC,s}$ and $p_{TA,s}$) according to the following equations:

$$\lambda_{a,s} = [\alpha\beta_a(A + \sigma A^*) + \beta_a(C + \sigma C^*)]K_S \qquad [1]$$

© 2024 Lin E et al. *JAMA Network Open.*

$$\beta_a = \frac{R_0}{d_R[\alpha p_{A,a} + P_{C,a}]K_S} + 0.0001a \qquad [2]$$

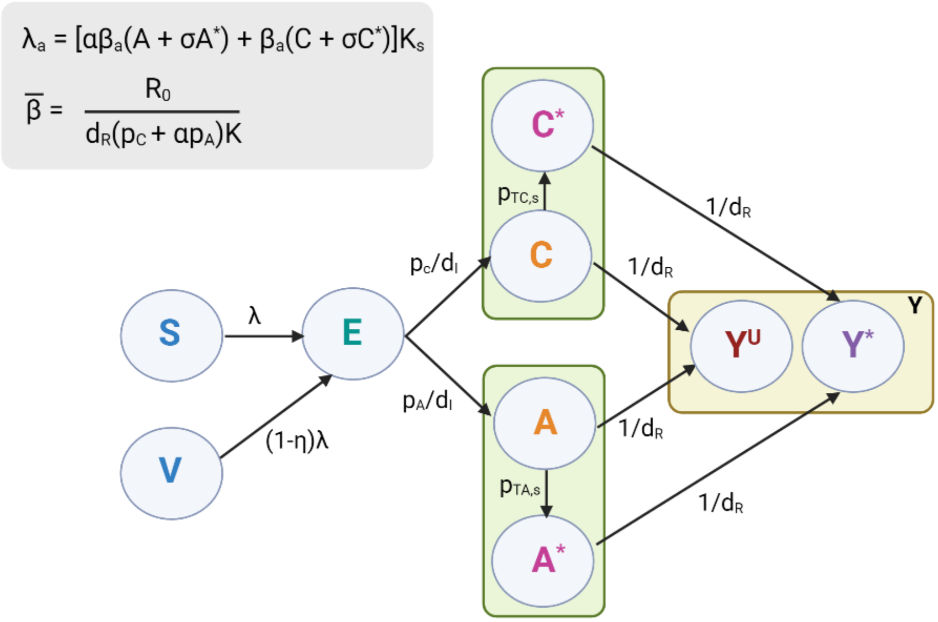


**eFigure 2.** SEIR Model Used to Simulate Data According to the Hypothesized Data Generating Mechanism. S = susceptible; V = vaccinated; E = exposed; C = clinical case; C* = reported clinical case; A = asymptomatic case; A* = reported asymptomatic case; Y = total recovered; $Y^U$ = unobserved total recovered; $Y^*$= observed total recovered. Because we simulate data over a short period of time (one semester), for this example, we assume some children start the semester vaccinated, and that no waning immunity will occur.

**eTable 1.** Parameter Values Used in the Simulation of Data

| Parameter | Definition | Value |
|---|---|---|
| $a$ | Age, in months, centered at 0 | Varied in simulation from -24 to 24 |
| $\lambda_{a,s}$ | School, and age-dependent force of infection | Calculated (equation 1) |
| $\beta_a$ | Age-dependent transmission rate of pathogen | Calculated (equation 2) |
| $R_0$ | Basic reproduction number | 2.5 |
| $\eta$ | Vaccine effectiveness | 0.5 |
| $d_I$ | Incubation period | 2 days |
| $d_R$ | Infectious period | 5 days |
| $p_{C,a}$ | Age-dependent probability case is clinical | 0.3 + 0.0001*$a$ |
| $p_{A,a}$ | Age-dependent probability case is asymptomatic | $1 - p_C$ |
| $\alpha$ | Relative infectivity of asymptomatic case to symptomatic case | 0.5 |
| $p_{TC,s}$ | School-dependent case ascertainment of clinical cases | $p_{TC,s=0} = 0.2$<br><br>$p_{TC,s=1}$ varied in simulation |
| $p_{TA,s}$ | School-dependent case ascertainment of asymptomatic cases | $p_{TA,s=0} = 0.05$<br><br>$p_{TA,s=1}$ varied in simulation |
| $K_S$ | School-dependent contact rates | $K_{S=0} = 10$<br><br>$K_{S=1}$ varied in simulation |
| $\sigma$ | Relative contribution of tested case to transmission | Varied between 0 (full isolation) and 1 (no isolation) |

© 2024 Lin E et al. *JAMA Network Open.*

Because we assumed that the primary contribution of schools to COVID-19 incidence is via $K_s, p_{TC,s}$, and $p_{TA,s}$, we first run the model for various combinations of these values at all age levels considered (here, we center age at 0 and examine within 24 months of this threshold). Specifically, we set the values equal to some baseline value for no school attendance ($S = 0$) and vary the degree that school attendance increases them. In cases where measurement is associated with the true outcome (i.e., tested cases are isolated), we assumed that reported cases contributed to the force of infection (via a parameter $\sigma < 1$) (eTable 2).

**eTable 2.** Scenarios From eFigure 1 Were Examined Using the Following Combinations of Parameters:

| **Scenario (eFigure 1)** | **Case detection** | **Testing ratio** *school detection to out of school detection* | **Removal of detected cases** |
|---|---|---|---|
| **A** | $p_{TC,s} = 1;\ p_{TA,s} = 1$ | Test ratio = 1 | No removal ($\sigma = 1$) |
| **B** | $p_{TC,s} < 1;\ p_{TA,s} < 1$ | Test ratio = 1 | $\sigma = 1$ |
| **C** | $p_{TC,s} < 1;\ p_{TA,s} < 1$ | Test ratio > 1 | $\sigma = 1$ |
| **D** | $p_{TC,s} < 1;\ p_{TA,s} < 1$ | Test ratio = 1 | $\sigma < 1$ |
| **E** | $p_{TC,s} < 1;\ p_{TA,s} < 1$ | Test ratio > 1 | $\sigma < 1$ |

We simulated observed cases in each age group, represented by $Y_a^*$, as well as true cases, represented by $Y_a = Y_a^* + Y_a^U$. We then fit the linear RD model to the simulated data using observed cases ($Y_a^*$) as the outcome, and true cases as the outcome ($Y_a$) to generate the true IRR and the observed IRR. Finally, we examined various functions of the testing ratio we should upweight the cases who did not attend school by such that an adjusted IRR approximates the true IRR.

Simulation study- Results

The true IRR was equivalent to the observed IRR over all variations of school-dependent contact rate and testing ratios in the scenario where case acquisition is not associated with the exposure (school attendance; DAGs A, B, and D in eFigure 1). Of note, the true and observed absolute difference (incidence rate difference as opposed to incidence rate ratio) would be differ in the presence of measurement error (D and D in eFigure 1).

Under the scenario where case detection is associated with the exposure but not associated with the true outcome (DAG C in eFigure 1), observed IRR exceeds true IRR. This bias is intuitive: higher case detections lead greater observed IRRs. In this context, the true IRR varies when school-dependent contact rates are varied, but not when the testing rate ratio is varied.

Under the scenario where case detection is associated with the exposure as well as with the true outcome (DAG E in eFigure 1), the observed IRR exceeds true IRR. However, the bias is more complex. At lower additional school-based contacts ($K$), the observed IRR at a high test ratio may be lower than the observed IRR at a low test ratio due to removal of infected individuals. At higher contact rates, the relationship flips (eFigure 3A, eFigure 4A-B). Of note, this complex relationship is only observed when case detection strongly prevents cases from contributing to the force of infection ($\sigma < 0.5$)

In both scenarios where the true IRR was not equal to the observed IRR, upweighting the number of cases in the population who did not attend school by the square root of the testing

© 2024 Lin E et al. *JAMA Network Open.*

ratio resulted in the best approximation of the adjusted IRR to the true IRR (eFigure 3A, eFigure 4A&C. Therefore, we used the square root of the testing rate ratio as our primary weighting factor in the main analysis.

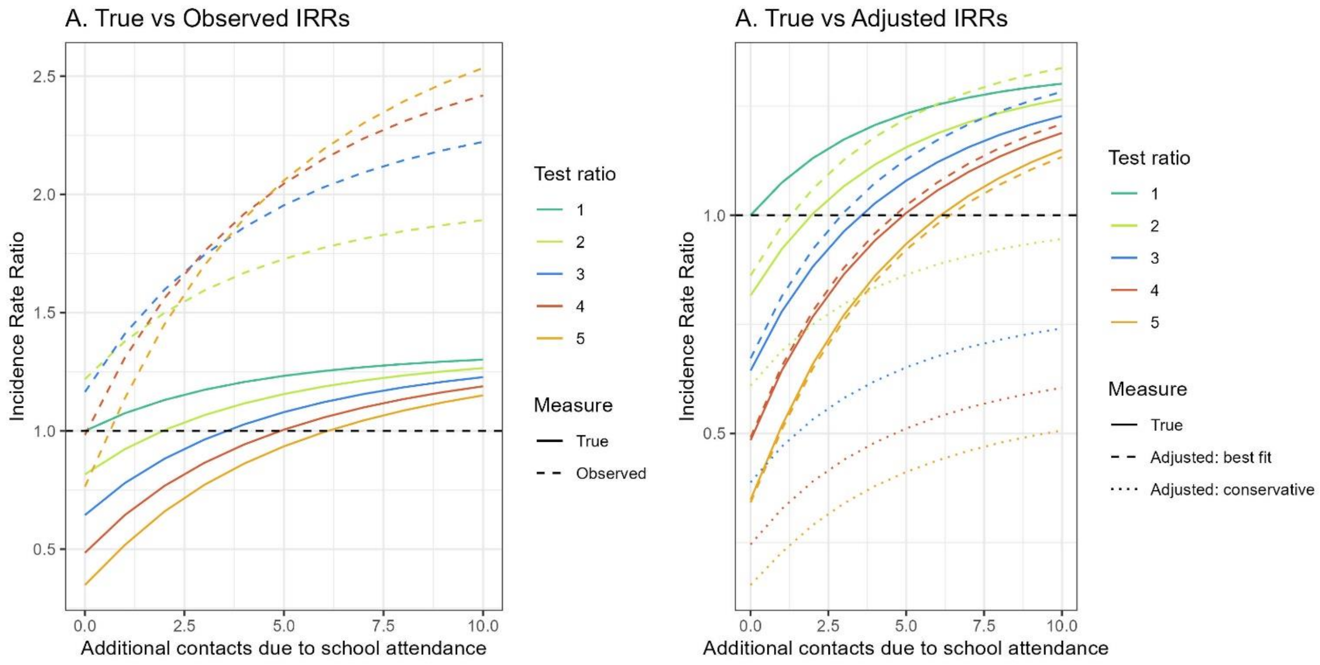


**eFigure 3.** Comparison of Observed and True Incidence Rate Ratios (A) and True and Adjusted Incidence Rate Rates (B). In this example, data is simulated according to the data generating mechanism depicted in eFigure 1E. In this mechanism, testing is positively associated with the exposure and reported outcome, but negative associated with the true outcome.

© 2024 Lin E et al. *JAMA Network Open.*

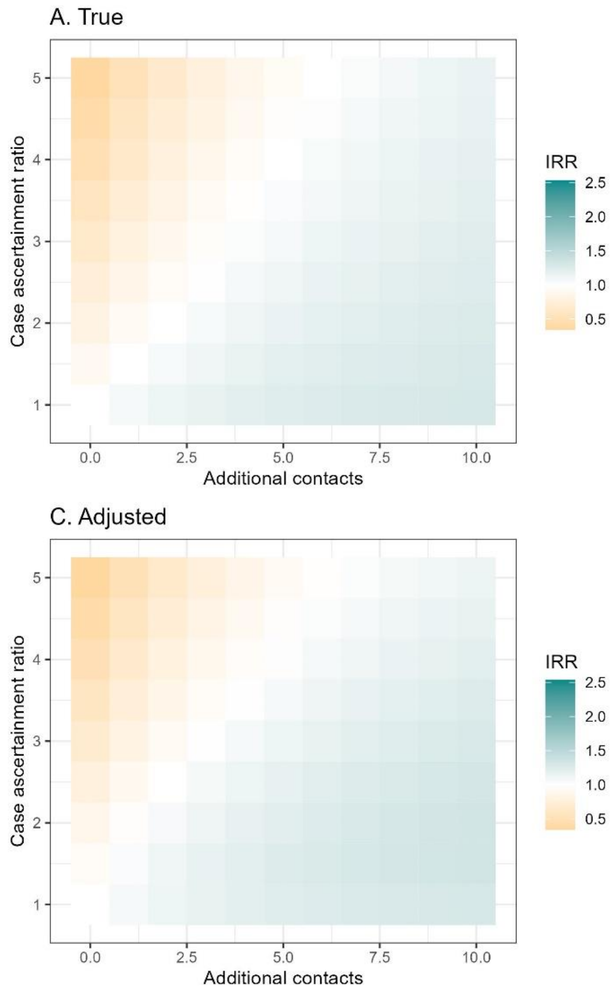


**eFigure 4.** Heat Map Comparison of Observed and True Incidence Rate Ratios (A) and True and Adjusted Incidence Rate Rates (B). In this example, data is simulated according to the data generating mechanism depicted in eFigure 1E. In this mechanism, testing is positively associated with the exposure and reported outcome, but negative associated with the true outcome.

© 2024 Lin E et al. *JAMA Network Open.*

Adjusting for testing bias

Testing effort was typically higher among school-aged children compared to younger children (eFigure 6). To contend with bias from differential case ascertainment, we weighted cases in the school-ineligible strata in order to approximate the number of cases that would have been observed in the school-ineligible age groups given equal testing effort (Supplemental Methods). To estimate these weights, we obtained aggregate data on the number of total and positive tests by county and age group (0- to 4-year-olds and 5- to 10- year-olds). Using population denominators from the American Community Survey,[1] we calculated the number of tests per age group and calculated the school-associated testing ratio as the testing rate among the school-eligible 5- to 10- year-olds versus 0- to 4- year-olds. These values ranged from 0.54 to 4.72, with a mean of 1.33 (eFigure 6A).

We weighted cases in the school-ineligible strata by the county's testing rate ratio in children 5- to 10- years-old vs. 0- to 4- years-old by the square root of the testing rate ratio for each county, according to our simulation analysis. In more conservative analyses, we weighted the school-ineligible strata by the testing rate ratio. This strategy is considered conservative because testing was conducted as a screening tool among asymptomatic populations, so each test administered had a lower probability of being a positive. Our simulation study further demonstrates that point.

We did not apply weights for the analyses with hospitalization as the outcome, as ascertainment of severe cases is more likely to be similar between populations.

© 2024 Lin E et al. *JAMA Network Open.*

Meta-analysis

We fit separate models for 46 of California's 58 counties, excluding 12 counties with few cases and births reported. We used a meta-analysis approach that has been commonly used to combine effect estimates across multiple locations, while permitting examination of effect heterogeneity.[2] We computed $\tau$ and the standard error on $\tau$ for each school period and county in California and used a fixed-effects meta-analysis to compute the pooled effect of $\tau$ for each school period.[2] The pooled effect was calculated as the weighted average of the individual county effects, where weights were equal to the inverse of the estimates' variances. In this way, more populous counties were generally assigned higher weights. We exponentiated the pooled estimate of $\tau$ to arrive at the pooled IRR.

To understand the effect of mitigation measures and other community-level factors on the impact of school attendance, we fit meta-regressions for each of the in-person semesters with predictors including county size,  population density,[1] racial/ethnicity composition, various measures of social vulnerability,[3] cumulative COVID-19 incidence at the start of the school semester (as a proxy for natural immunity),[4] vaccination coverage at the start of the school semester,[5] and the percent of survey respondents who reported never wearing a mask[6] (eTable 5). Predictors were tested in univariate models and in models adjusting for total county population. We also fit one meta-regression with all in-school periods, adding a fixed effects for semester and univariate predictors described.

© 2024 Lin E et al. *JAMA Network Open.*

Sensitivity Analyses

To assess the robustness of results to model specification, we ran analyses varying bandwidth ($h$) from 8 to 24 months and testing three different functional bases for the relationship of age ($x_i$) to COVID-19 outcomes and its modification by school eligibility. These included linear and quadratic models, as well as generalized additive models in which locally linear, or LOESS, curves were used to relate age to the outcome.[7] For the local linear regression, we did not include an interaction term, as the LOESS fit should be flexible enough to capture functional differences in the relationship between age and COVID-19 outcomes above and below the school age-eligibility threshold. We compared model fit using the Akaike Information Criterion (AIC), a method for model selection that allows for comparison of likelihood-based goodness-of-fit metrics, while penalizing for additional model parameters.[8,9]

Power Simulation

We conducted a power analysis to determine the minimum effect size for the relationship between school attendance and hospitalizations at which we would have 80% power and 95% confidence. Following the approach outlined by Bilinski and Hatfield,[10] we first fit the Poisson regression discontinuity model outlined in equation 1 (main text), using hospitalizations as the outcome and assuming a linear relationship between age and hospitalizations. We extracted model residuals, $u_i$, and generated predicted values, $\widehat{p}_i$ , from this model, setting $Z_i$ equal to 0 to simulate no effect of school eligibility.

© 2024 Lin E et al. *JAMA Network Open.*

We then generated 10,000 synthetic data sets using the following data-generating process. For each of the 10,000 iterations, we drew a value for $\log(\widehat{y_i})$ from a normal distribution centered at:

$$\log(\hat{y}_i) = \hat{p}_i + \tau Z_i$$

and with standard errors equal to the squared model residuals. We examined values for $\tau$ where $\tau \in \{0.6, 1.2\}$.

We then re-fit the Poisson regression discontinuity model to the simulated datasets, and determined whether the effect estimate on $\tau$ was statistically significant for a significance level of 0.95. Power was calculated as the proportion of the 10,000 simulations where the effect estimate was significant at the 95% confidence level. We exponentiated $\tau$ to observe the IRRs at which we had 80% power.

© 2024 Lin E et al. *JAMA Network Open.*

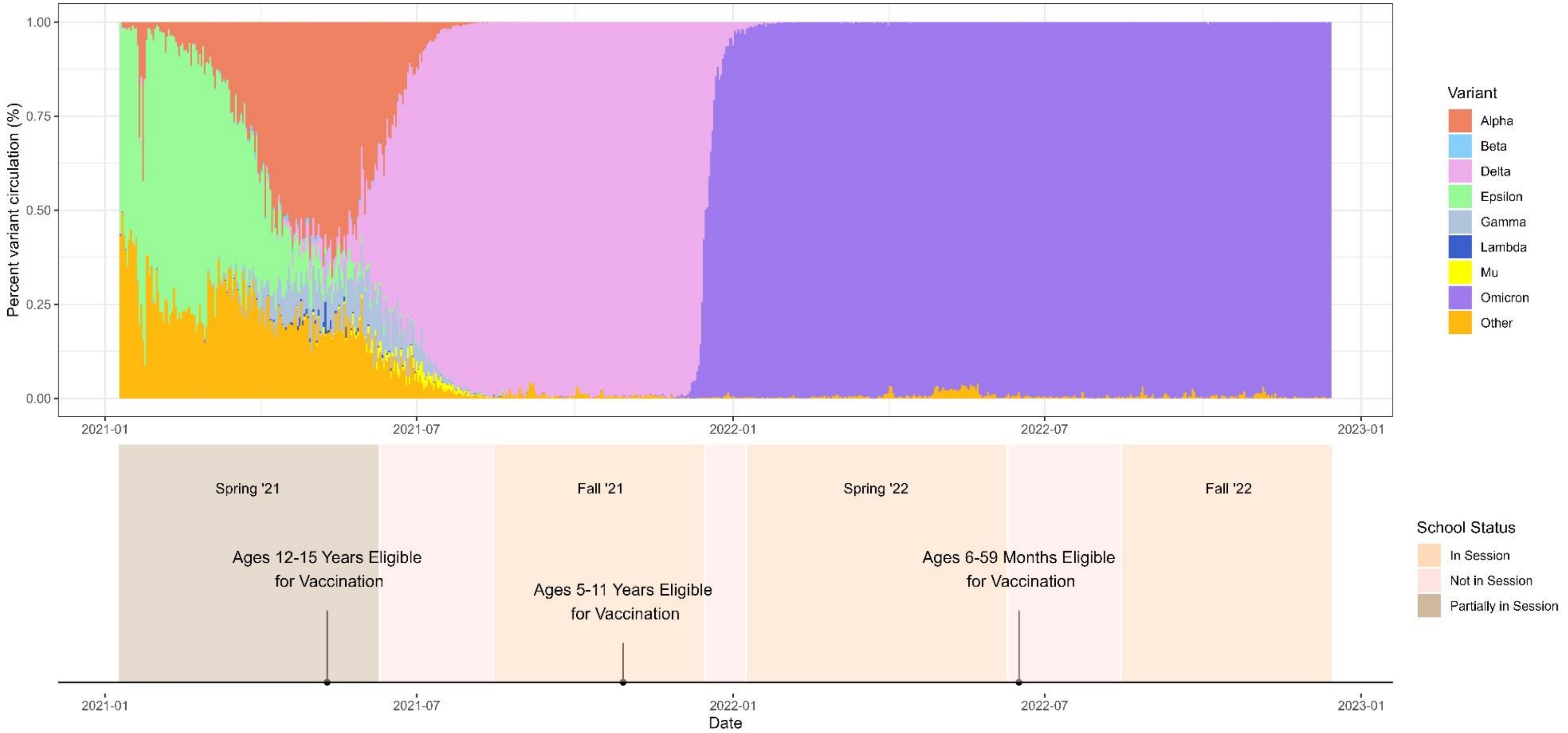


**eFigure 5.** Timeline of Circulating SARS-CoV-2 Variants, Vaccination Approval Dates, and School Periods. Variant data from [11]

© 2024 Lin E et al. *JAMA Network Open.*

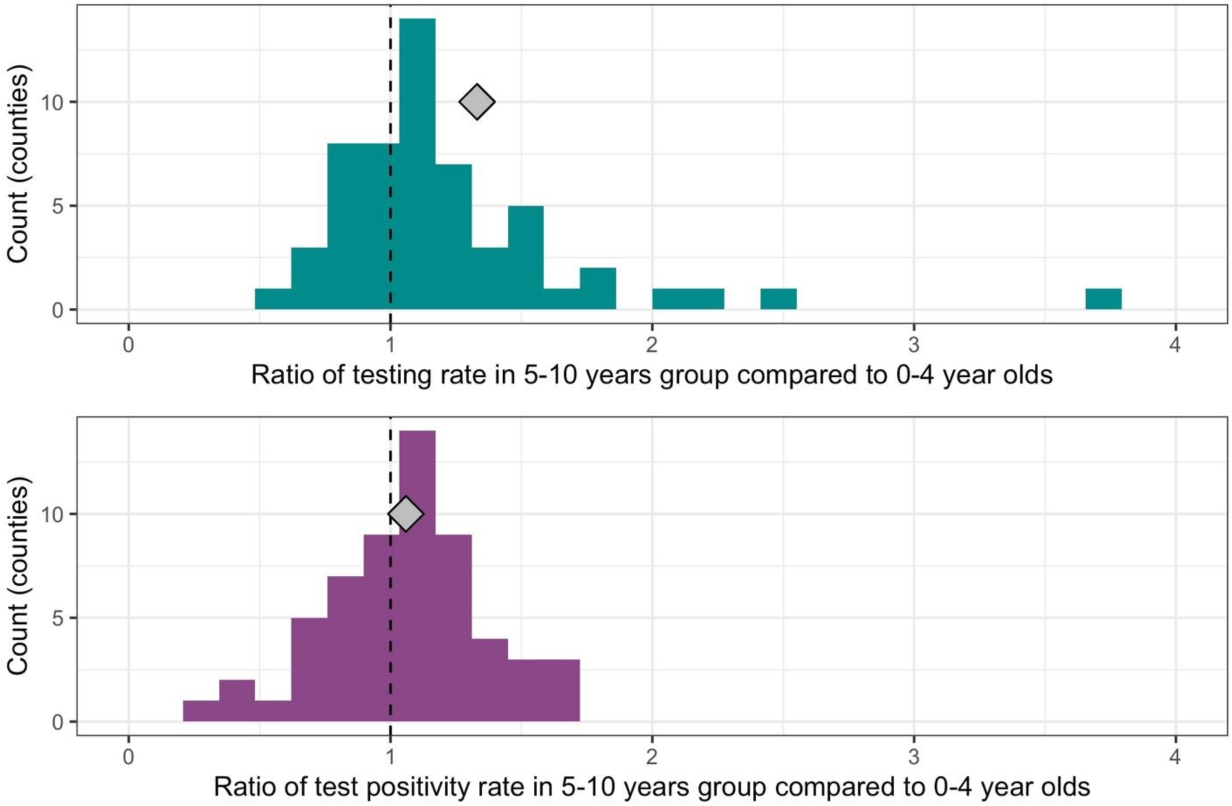


**eFigure 6.** Distribution of Testing Rate Ratios and Test Positivity Rate Ratios Among California Counties Comparing Testing Rates and Test Positivity Rates Among School-Aged Children (5-10 Years) to Non-School-Aged Children (0-4 Years). **Vertical line indicates event testing and test positivity rate, and gray diamonds symbolize the mean ratio.**

© 2024 Lin E et al. *JAMA Network Open.*

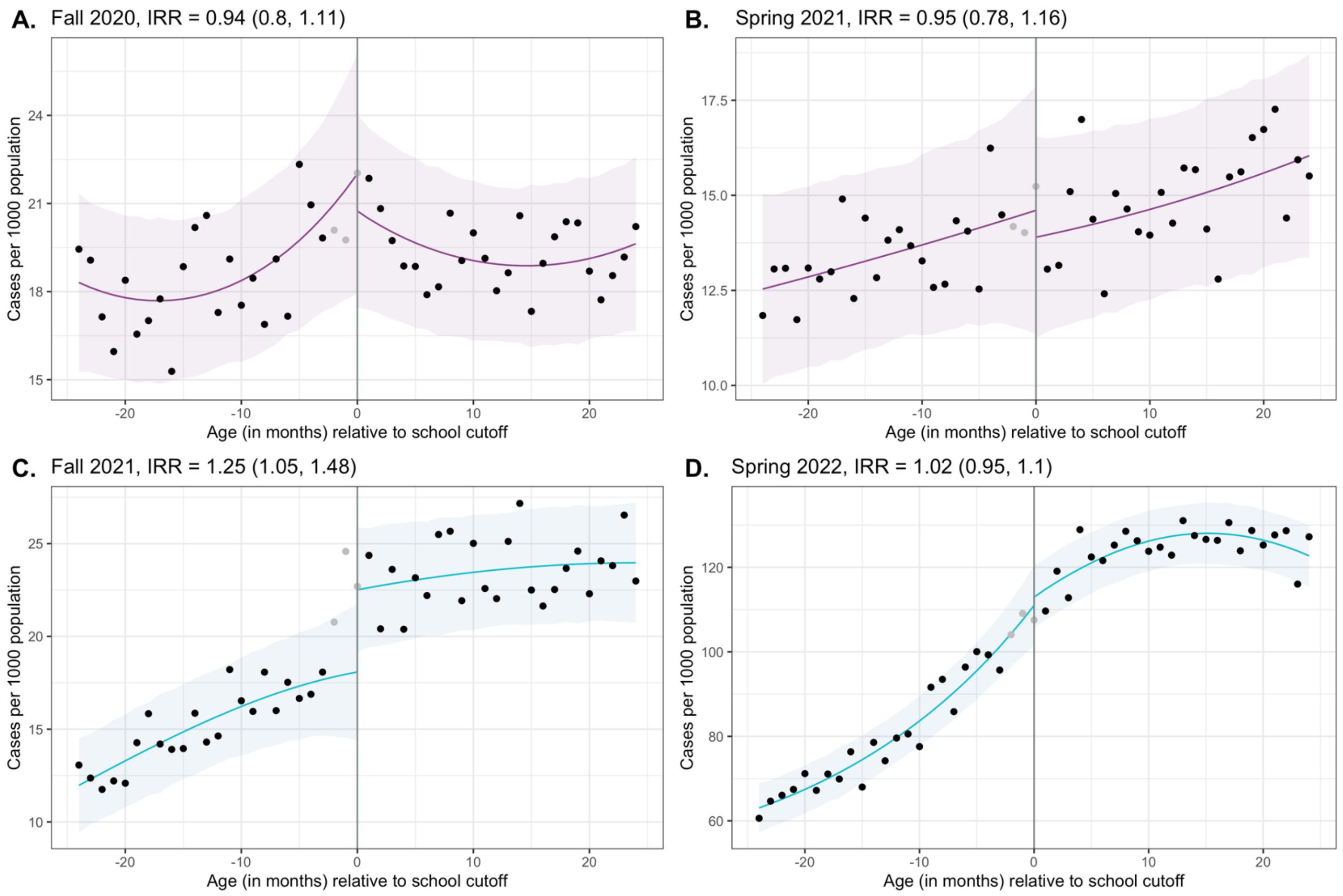


**eFigure 7.** COVID-19 Incidence as a Function of Child Age Relative to the September 1st Cutoff for Elementary School Attendance, for Models Assuming a Quadratic Relationship Between Age and Incidence. Ages right of the threshold indicate that the child is age-eligible to attend elementary school (K-5). Model fits are shown for the fall (A) and spring (B) semesters when school was remote (purple colors) and during the fall (C) and spring (D) semesters when school was in-person (blue colors). Black dots indicate observed data, lines indicate model fit, and shaded region indicates 95% prediction intervals. For this example, weighting to adjust for testing biases is not performed.Plots shown are selected from Los Angeles County, the most populous county in California, and models shown use a bandwidth of 24 months and drop children eligible for transitional kindergarten (TK).

© 2024 Lin E et al. *JAMA Network Open.*

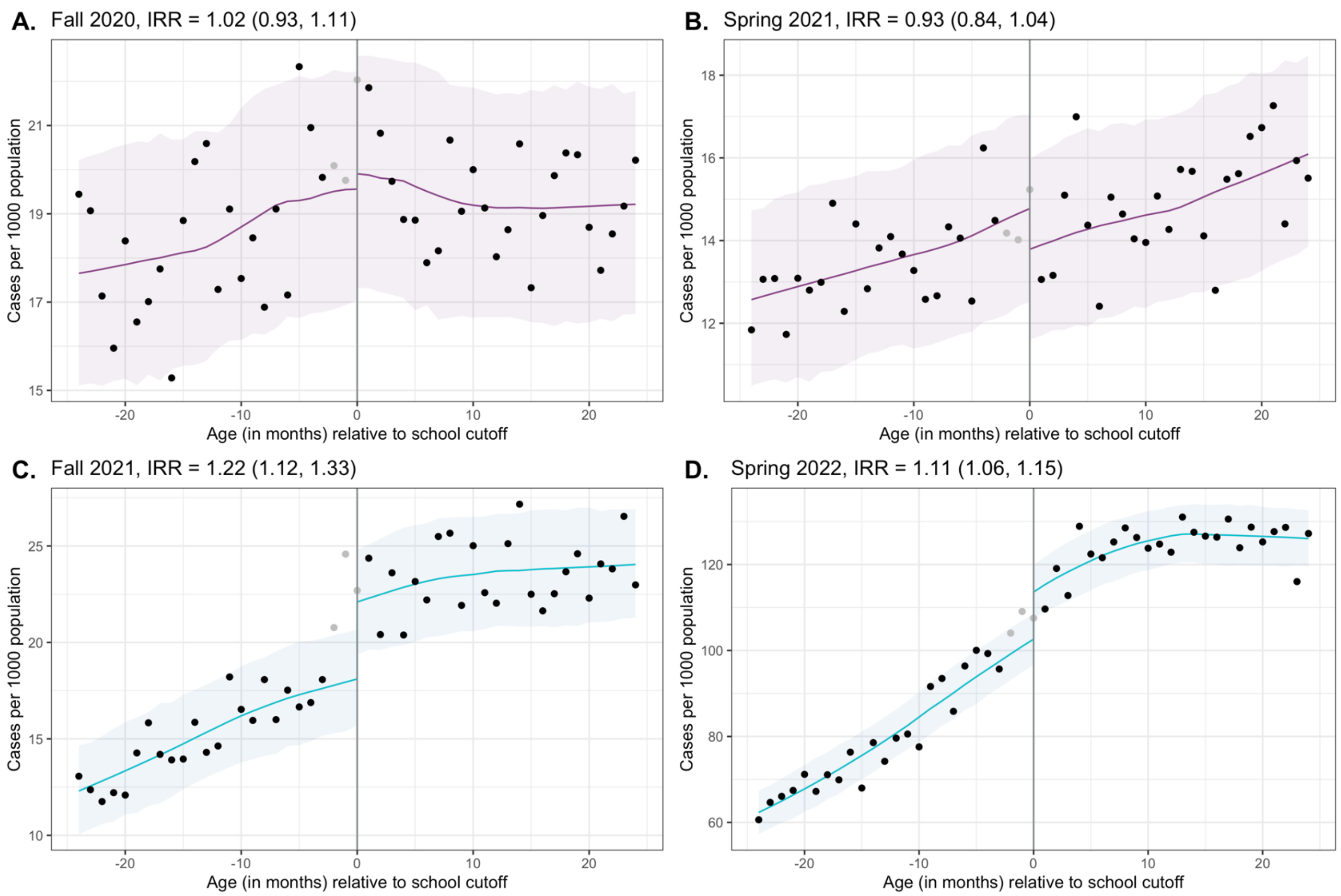


**eFigure 8.** COVID-19 Incidence as a Function of Child Age Relative to the September 1st Cutoff for Elementary School Attendance, for Models That Use Local Linear Regression. Ages right of the threshold indicate that the child is age-eligible to attend elementary school (K-5). Model fits are shown for the fall (A) and spring (B) semesters when school was remote (purple colors) and during the fall (C) and spring (D) semesters when school was in-person (blue colors). Black dots indicate observed data, lines indicate model fit, and shaded region indicates 95% prediction intervals. For this example, weighting to adjust for testing biases is not performed. Plots shown are selected from Los Angeles County, the most populous county in California, and models shown use a bandwidth of 24 months and drop children eligible for transitional kindergarten (TK).

© 2024 Lin E et al. *JAMA Network Open.*

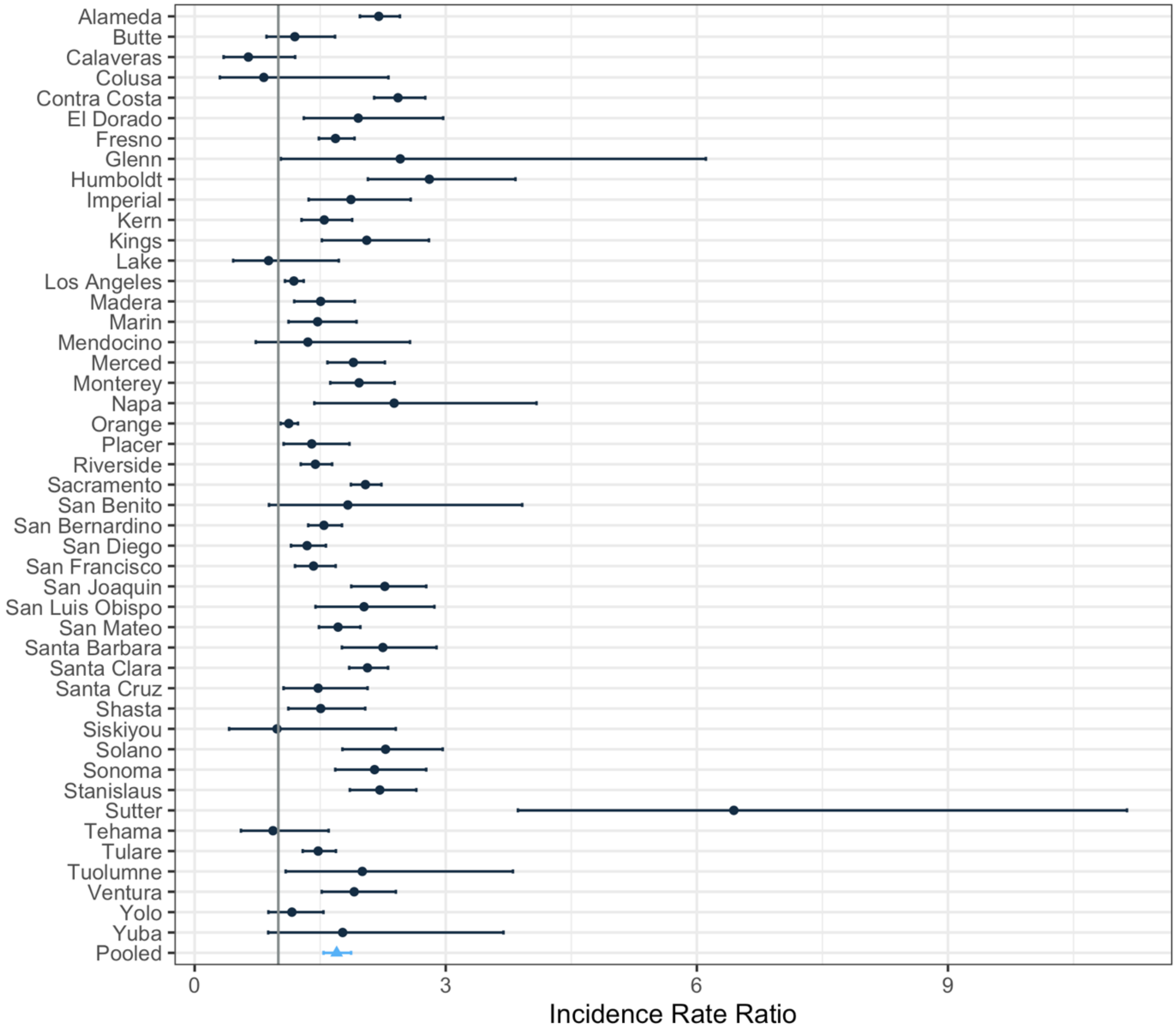


**eFigure 9.** County-Specific (Black) and Pooled (Blue) Incidence Rate Ratios (IRR) Representing the Incidence of COVID-19 in the Fall 2021 Semester of the 2021-2022 Academic Year Among Children Born Just Before the Age-Eligibility Threshold for Elementary School Compared to Those Born Just After. Results are for linear models with a bandwidth of 24 months, dropping children eligible for transitional kindergarten (TK).

© 2024 Lin E et al. *JAMA Network Open.*

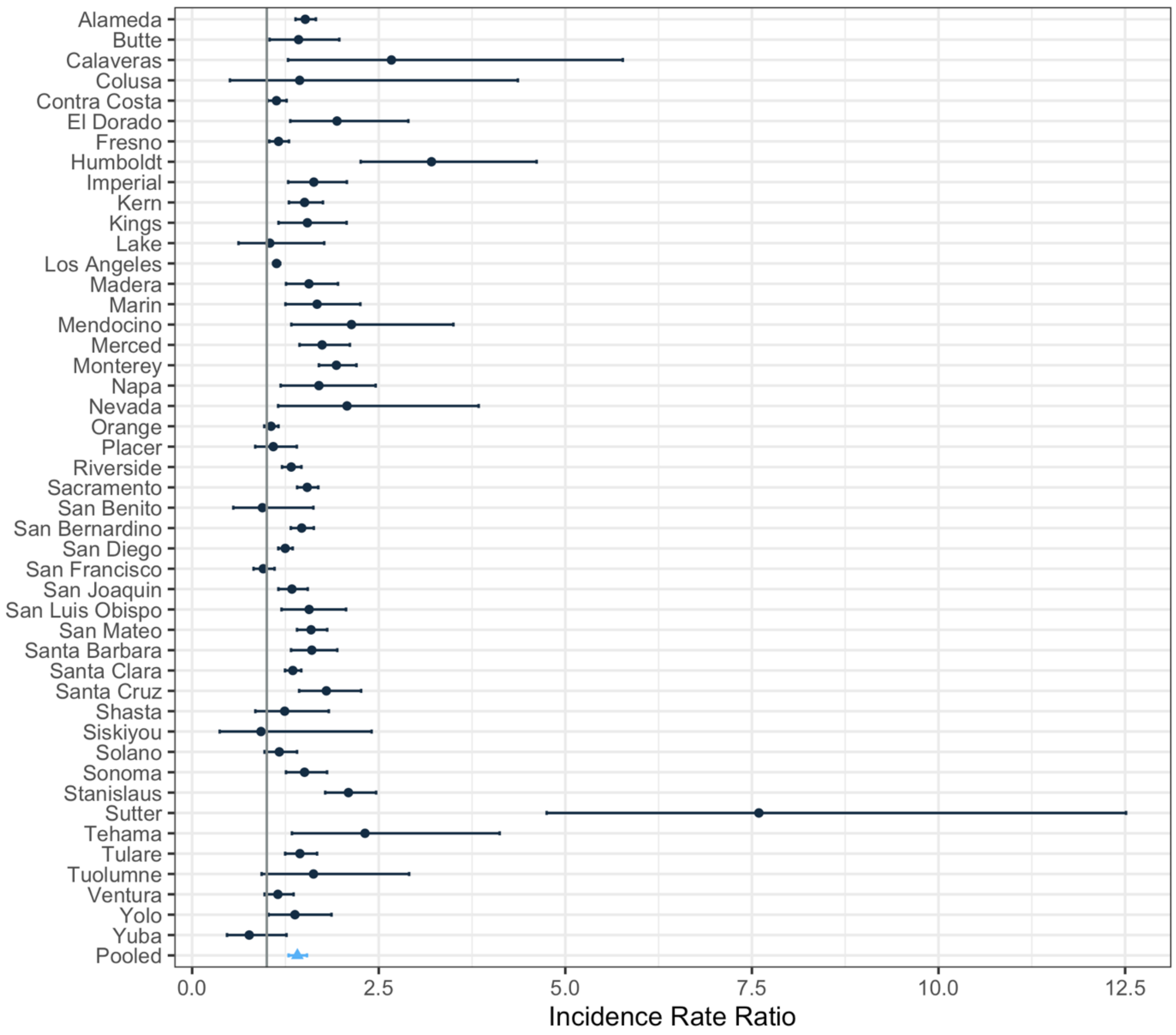


**eFigure 10.** County-Specific (Black) and Pooled (Blue) Incidence Rate Ratios (IRR) Representing the Incidence of COVID-19 in the Spring 2022 Semester of the 2021-2022 Academic Year Among Children Born Just Before the Age-Eligibility Threshold for Elementary School Compared to Those Born Just After. **Results are for linear models with a bandwidth of 24 months, dropping children eligible for transitional kindergarten (TK).**

© 2024 Lin E et al. *JAMA Network Open.*

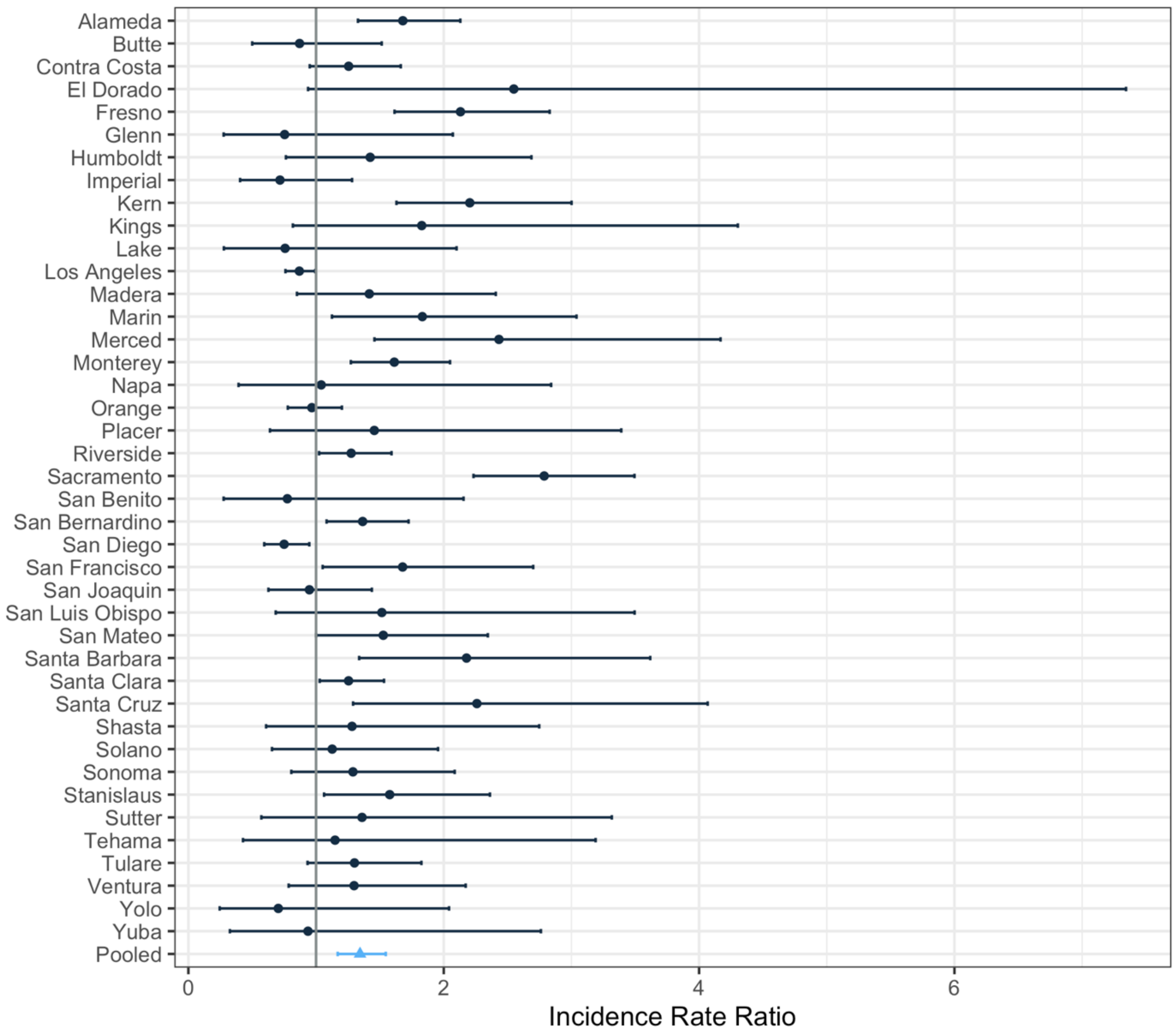


**eFigure 11.** County-Specific (Black) and Pooled (Blue) Incidence Rate Ratios (IRR) Representing the Incidence of COVID-19 in the Fall 2022 Semester of the 2021-2022 Academic Year Among Children Born Just Before the Age-Eligibility Threshold for Elementary School Compared to Those Born Just After**. Results are for linear models with a bandwidth of 24 months, dropping children eligible for transitional kindergarten (TK).**

© 2024 Lin E et al. *JAMA Network Open.*

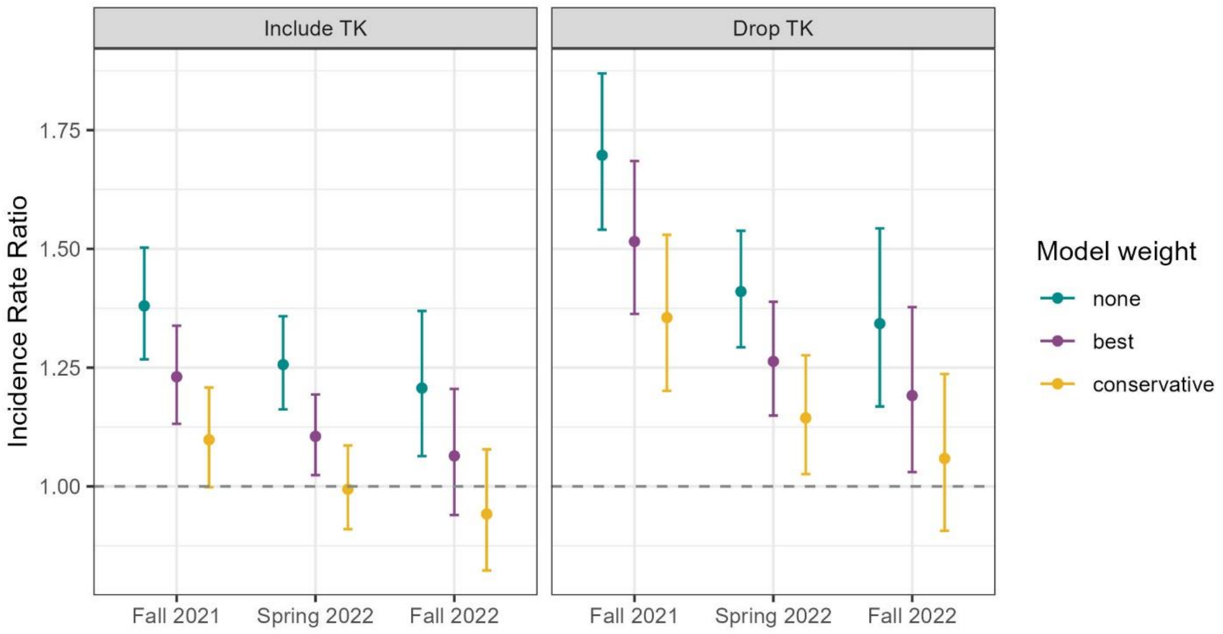


eFigure 12. Comparison of IRRs During In-School Periods Adjusting for Testing Biases and Not Adjusting for Testing Differences. Results are for linear models with a bandwidth of 24 months.

© 2024 Lin E et al. *JAMA Network Open.*

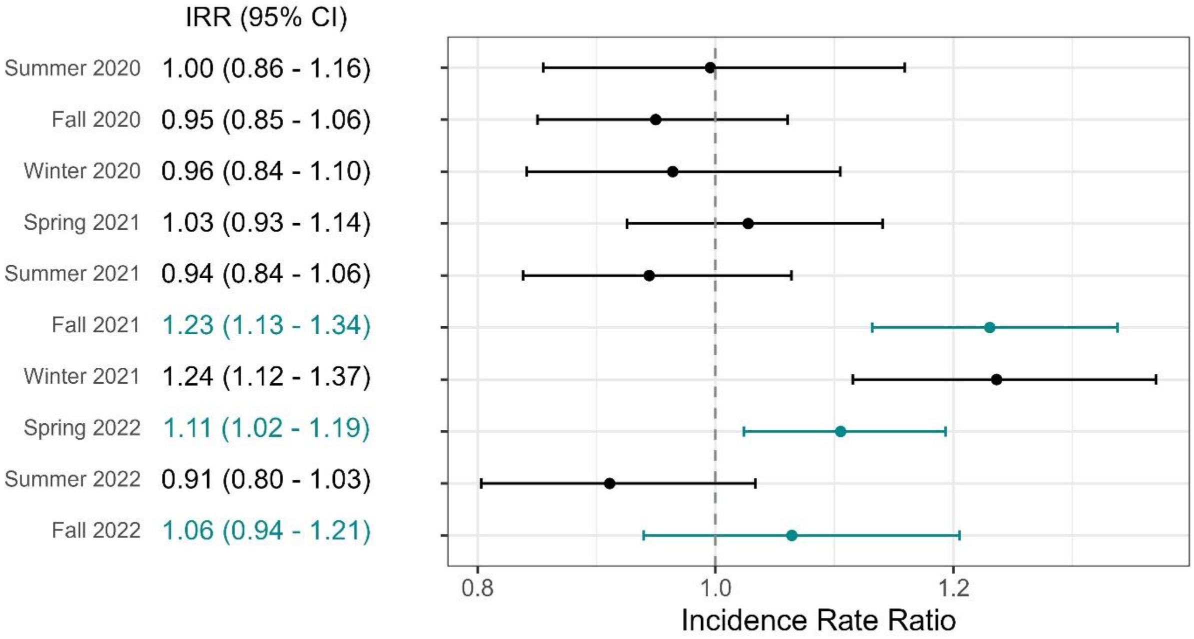


eFigure 13. Pooled Incidence Rate Ratios (IRR) Representing the Incidence of COVID-19 Among Children Born Just Before the Age-Eligibility Threshold for Elementary School Compared to Those Born Just After. Results are for linear models with a bandwidth of 24 months, including children eligible for transitional kindergarten (TK), and using the best weighting adjustment.

© 2024 Lin E et al. *JAMA Network Open.*

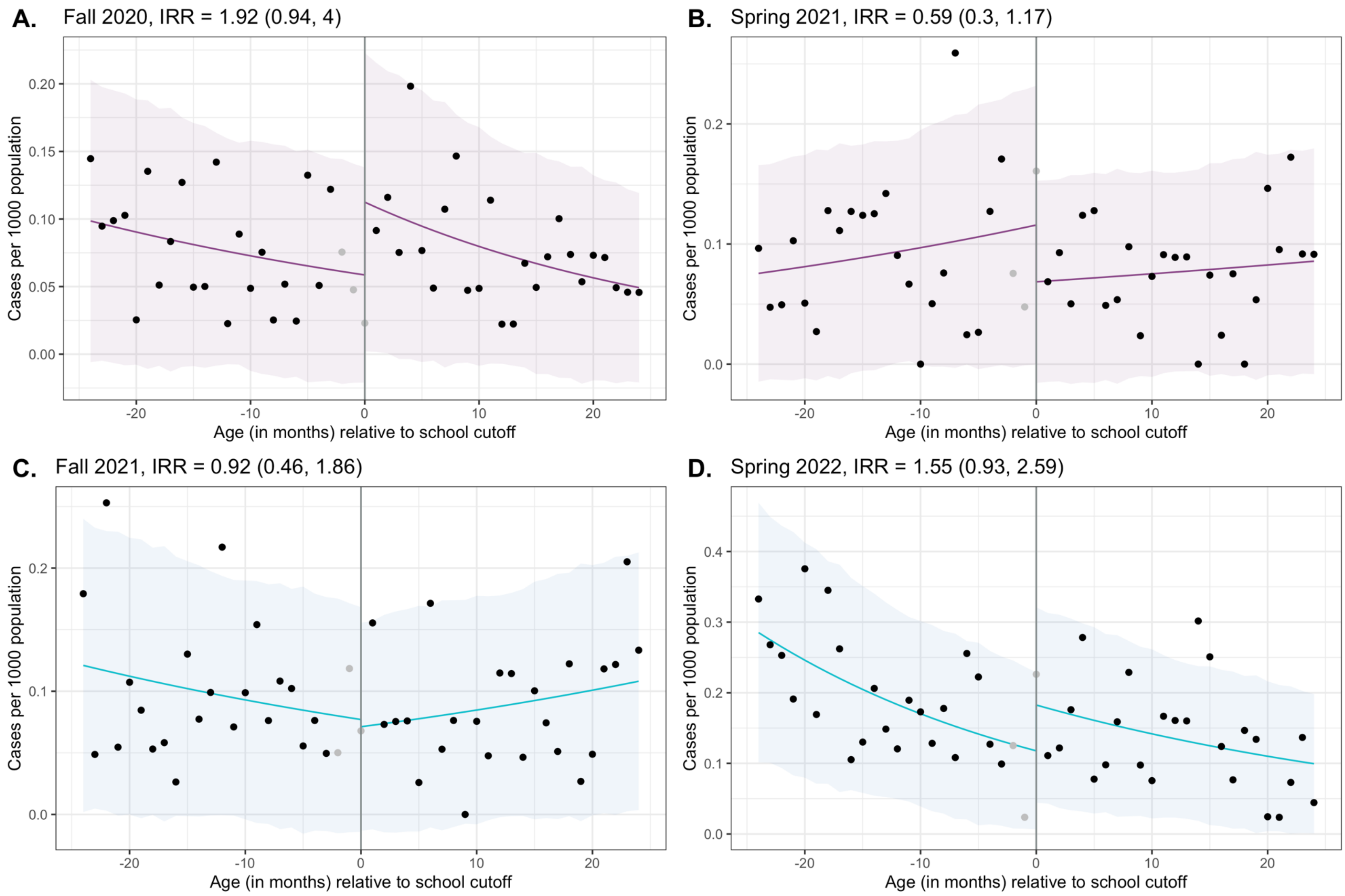


eFigure 14. COVID-19 Hospitalization as a Function of Child Age Relative to the September 1st Cutoff for Elementary School Attendance, for Models Assuming a Linear Relationship Between Age and Hospitalizations. Ages right of the threshold indicate that the child is age-eligible to attend elementary school (K-5). Model fits are shown for the fall (A) and spring (B) semesters when school was remote (purple colors) and during the fall (C) and spring (D) semesters when school was in-person (blue colors). Black dots indicate observed data, lines indicate model fit, and shaded region indicates 95% prediction intervals. Models shown use a bandwidth of 24 months and drop children eligible for transitional kindergarten (TK).

© 2024 Lin E et al. *JAMA Network Open.*

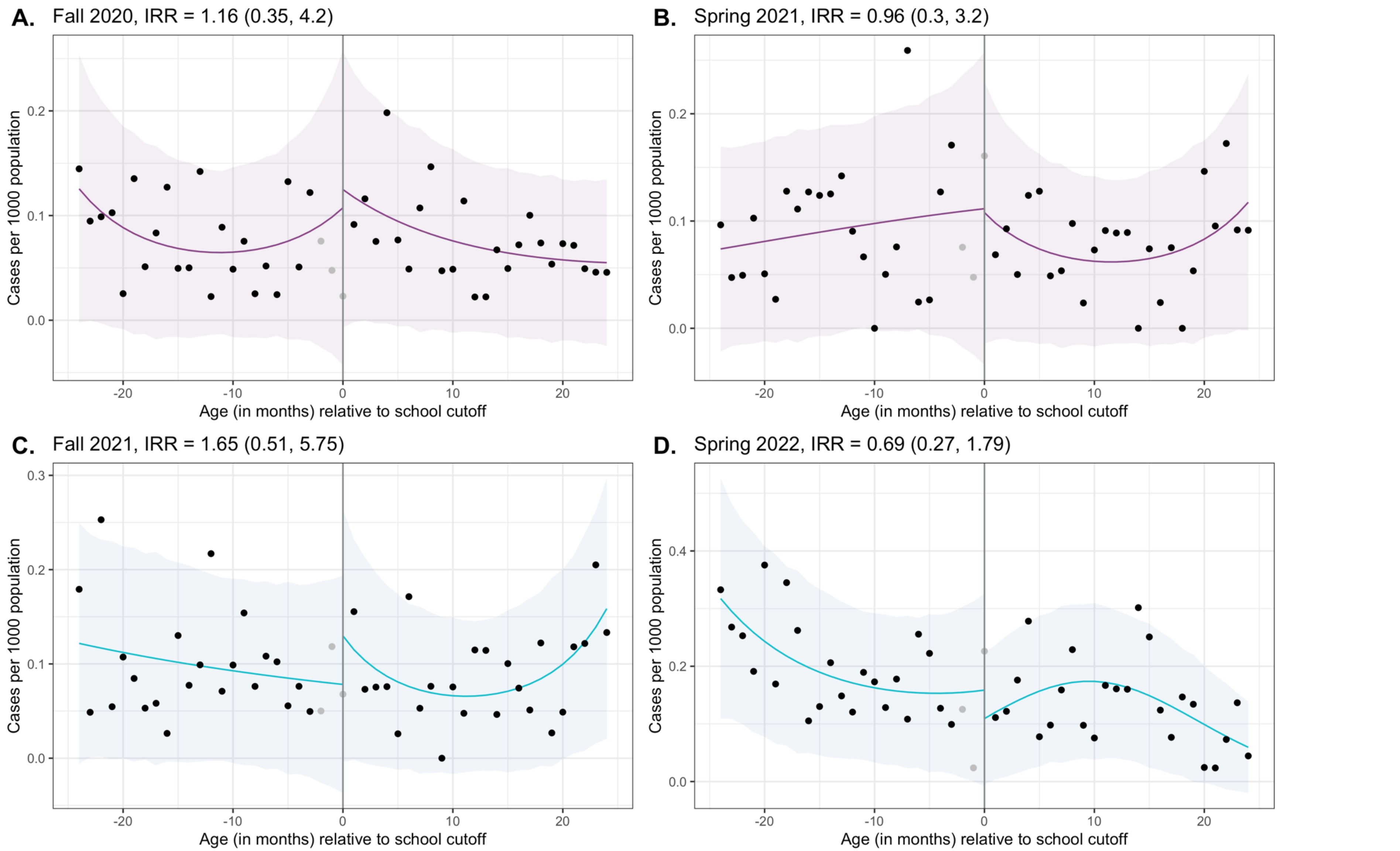


eFigure 15. COVID-19 Hospitalization as a Function of Child Age Relative to the September 1st Cutoff for Elementary School Attendance, for Models Assuming a Quadratic Relationship Between Age and Hospitalizations. Ages right of the threshold indicate that the child is age-eligible to attend elementary school (K-5). Model fits are shown for the fall (A) and spring (B) semesters when school was remote (purple colors) and during the fall (C) and spring (D) semesters when school was in-person (blue colors). Black dots indicate observed data, lines indicate model fit, and shaded region indicates 95% prediction intervals. Models shown use a bandwidth of 24 months and drop children eligible for transitional kindergarten (TK).

© 2024 Lin E et al. *JAMA Network Open.*

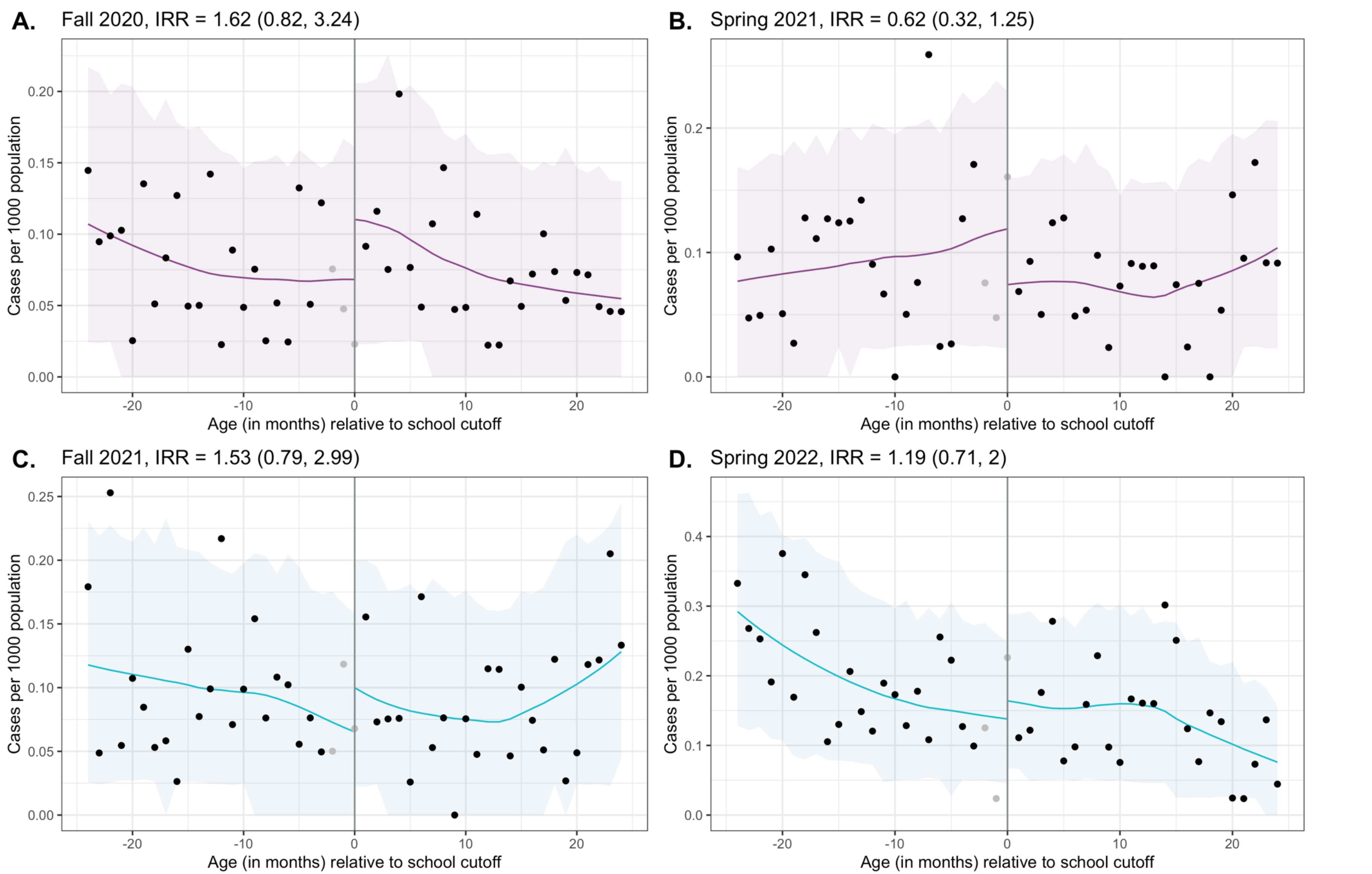


eFigure 16. COVID-19 Hospitalization as a Function of Child Age Relative to the September 1st Cutoff for Elementary School Attendance, for Models That Use Local Linear Regression. Ages right of the threshold indicate that the child is age-eligible to attend elementary school (K-5). Model fits are shown for the fall (A) and spring (B) semesters when school was remote (purple colors) and during the fall (C) and spring (D) semesters when school was in-person (blue colors). Black dots indicate observed data, lines indicate model fit, and shaded region indicates 95% prediction intervals. Models shown use a bandwidth of 24 months, and drop children eligible for transitional kindergarten (TK).

© 2024 Lin E et al. *JAMA Network Open.*

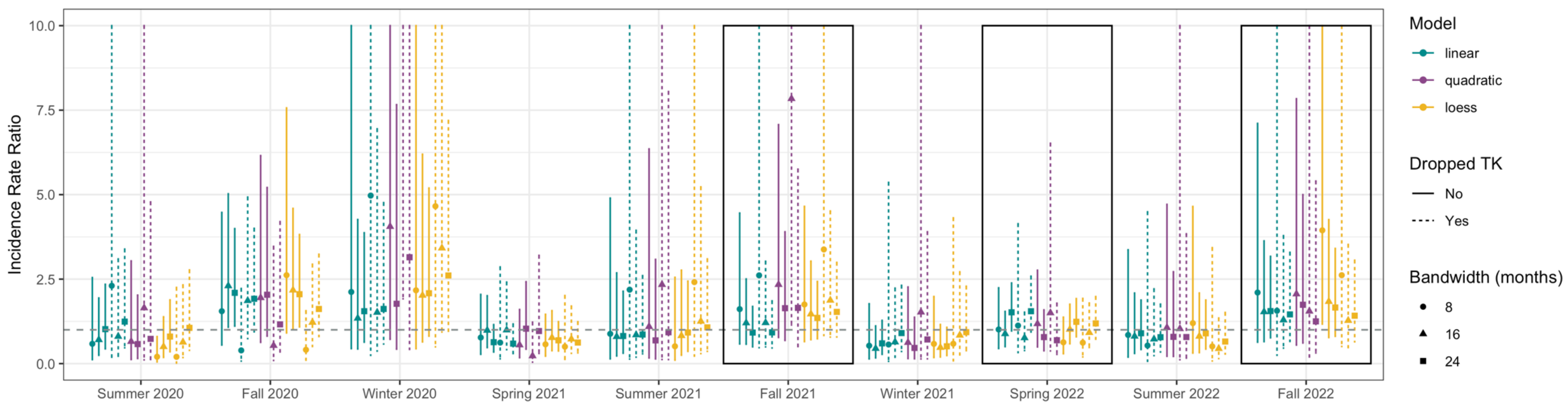


eFigure 17. Associations Between Elementary School Age-Eligibility and Hospitalizations for COVID-19 by School Period and Model Parameterization. We examined the sensitivity of results various model parametrizations (e.g., local linear regression (loess), linear relationship between age and outcome, and quadratic relationship between age and outcome) and bandwidths. We also considered the effect of excluding individuals born between September and November, as these individuals were eligible for transitional kindergarten (TK). In-person semesters are outlined in black boxes.

© 2024 Lin E et al. *JAMA Network Open.*

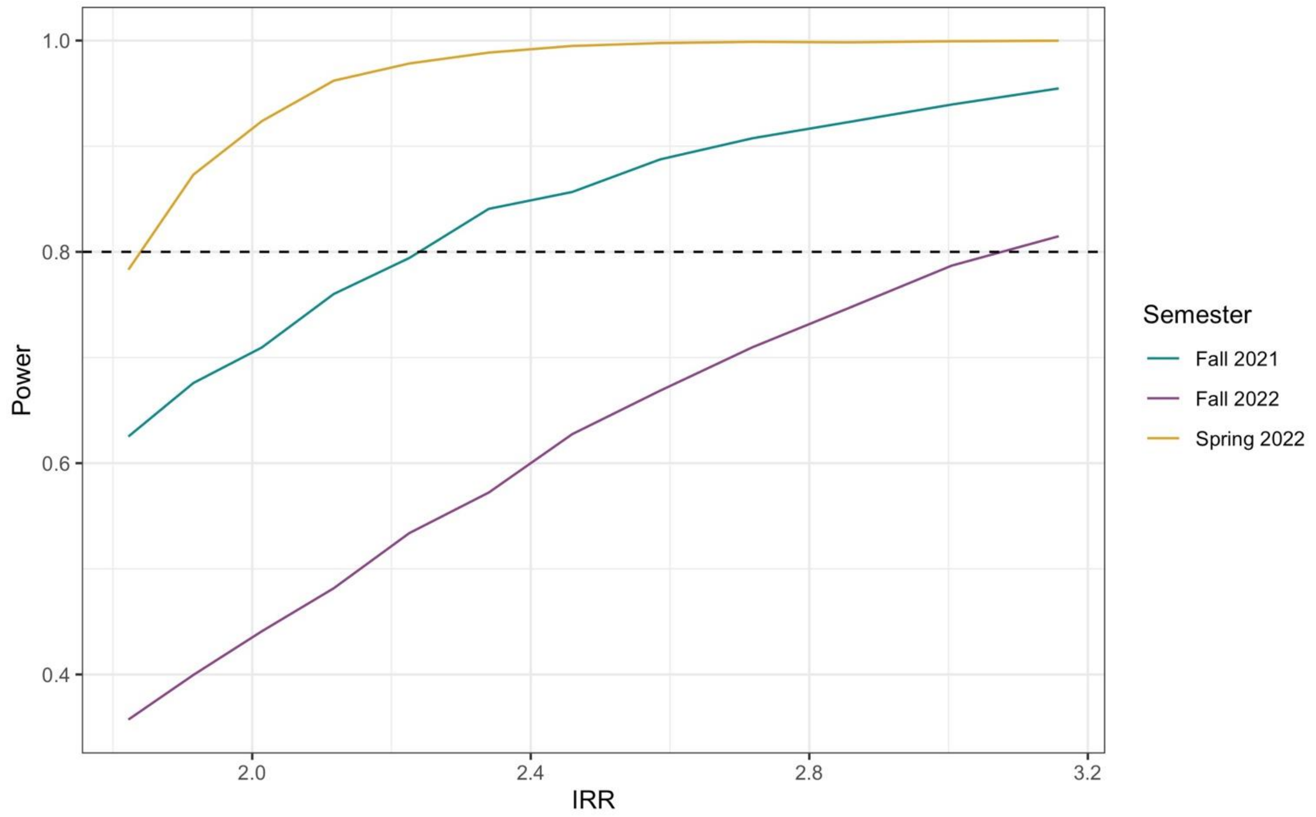


eFigure 18. Power Analysis for the Association Between Hospitalizations and School Eligibility. Graph displays the power associated with various effect sizes (incidence rate ratios, IRRs) for the three in-person semesters examined. In our power analysis, we determined that the minimum effect sizes for which we had 80% power were 2.2 (fall 2021), 1.9 (spring 2022), and 3.1 (fall 2022).

© 2024 Lin E et al. *JAMA Network Open.*

eTable 3. Total Number of Cases and Hospitalizations Among the Subsample of Children Who Fell Within 24 Months, in Either Direction, of the Elementary School Attendance Threshold. Bolded rows indicate periods when schools had in-person instruction. For example, for the 2020/2021 academic year, children born before September 1, 2015, would be eligible for kindergarten. Thus, for a bandwidth of 24, children born no more than 24 months before or after September 1, 2015, and who experienced a confirmed COVID-19 infection within the 2020/2021 academic year, would be included in our subsample.

| Academic year | Semester investigated | Date ranges | Birthdate cutoff | Total cases | Total hospitalizations |
|---|---|---|---|---|---|
| 2019/2020 | Summer | May 16, 2020 – August 14, 2020 | Sept 1, 2014 | 16,647 | 87 |
| 2020/2021 | Fall | August 15, 2020 – December 14, 2020 | Sept 1, 2015 | 51,361 | 151 |
| 2020/2021 | Winter | December 15, 2020 – January 14, 2021 | Sept 1, 2015 | 28,650 | 81 |
| 2020/2021 | Spring | January 15, 2021 – May 15, 2021 | Sept 1, 2015 | 59,805 | 170 |
| 2020/2021 | Summer | May 16, 2021 – August 14, 2021 | Sept 1, 2015 | 36,316 | 80 |
| **2021/2022** | **Fall** | August 15, 2021 – December 14, 2021 | **Sept 1, 2016** | **134,919** | **183** |
| 2021/2022 | Winter | December 15, 2021 – January 14, 2022 | Sept 1, 2016 | 83,327 | 111 |
| **2021/2022** | **Spring** | January 15, 2022 – May 15, 2022 | **Sept 1, 2016** | **212,093** | **318** |
| 2021/2022 | Summer | May 16, 2022 – August 14, 2022 | Sept 1, 2016 | 37,072 | 121 |
| **2022/2023** | **Fall** | August 15, 2022 – December 14, 2022 | **Sept 1, 2017** | **28,088** | **121** |

© 2024 Lin E et al. *JAMA Network Open.*

eTable 4. Incidence Rate Ratios (IRRs) and 95% Confidence Intervals Comparing the Incidence of COVID-19 Among Children Born Just Before the Threshold for Elementary School Attendance (September 1st) Compared to Just After. Rows highlighted in blue are the three in-person semesters included in the study period. Children born between September 2nd and December 1st are eligible for transitional kindergarten (TK), so we conducted analyses both excluding and including children born in September – November. Testing-adjusted estimates attempt to correct for testing differences between schooled and unschooled populations. Results for non in-school periods are the same as in Table 1 in the Main Text.

| | **Excluding TK-eligible children IRR (95% CI)** | | | **Including TK-eligible children IRR (95% CI)** | | |
|---|---|---|---|---|---|---|
| School period | Testing-adjusted: Conservative | Testing-adjusted: Best fit | Not adjusted | Testing-adjusted: Conservative | Testing-adjusted: Best fit | Non adjusted |
| Fall 2021 | 1.36 (1.20 - 1.53) | 1.52 (1.36 - 1.68) | 1.70 (1.54 - 1.87) | 1.10 (1.00 - 1.21 | 1.23 (1.13 - 1.34) | 1.38 (1.50 - 1.27) |
| Spring 2022 | 1.14 (1.03 - 1.28) | 1.26 (1.15 - 1.39) | 1.41 (1.29 - 1.54) | 0.99 (0.91 - 1.09) | 1.11 (1.02 - 1.19) | 1.26 (1.16 - 1.36) |
| Fall 2022 | 1.06 (0.91 - 1.24) | 1.19 (1.03 - 1.38) | 1.34 (1.17 - 1.55) | 0.94 (0.82 - 1.08) | 1.06 (0.94 - 1.21) | 1.21 (1.06 - 1.37) |

*point-estimates and associated CI's should be interpreted as approximations that attempt to adjust for bias rather than as completely bias-free

© 2024 Lin E et al. *JAMA Network Open.*

eTable 5. Results of Meta-Analysis. Values represent the ratio of county-specific incidence rate ratios (representing the association between age-eligibility for elementary school and incidence) for a given unit change in county-level predictor

| **Variable [source]** | **Unit change in predictor** | **Fall 2021-2022** Ratio (95% CI) | **Spring 2021-2022** Ratio (95% CI) | **Fall 2022-2023** Ratio (95% CI) |
|---|---|---|---|---|
| County population [1] | 1 million persons | 0.95 (0.91 - 1.00) | 0.97 (0.94 - 1.00) | 0.94 (0.89 - 1.00) |
| Population density [1] | 1000 persons per square mile | 0.96 (0.89 - 1.04) | 0.92 (0.86 - 0.99) | 1.00 (0.88 - 1.14) |
| Percentage (%) of people reporting never using a mask [6] | 10 percentage points | 1.19 (0.68 - 2.08) | 1.18 (0.68 - 2.04) | 1.09 (0.42 - 2.85) |
| Vaccination coverage at the start of the semester in children aged 5-11 years [5] | 10 additional vaccines per 100 persons | 1.05 (0.95 - 1.16) | 0.98 (0.93 - 1.04) | 1.00 (0.92 - 1.08) |
| Vaccination coverage at the start of the semester in children aged 12-17 years [5] | 10 additional vaccines per 100 persons | 1.04 (0.98 - 1.01) | 0.97 (0.92 - 1.02) | 0.97 (0.90 - 1.06) |
| Vaccination coverage at the start of the semester in adults aged 18-49 years [5] | 10 additional vaccines per 100 persons | 1.03 (0.97 - 1.10) | 0.96 (0.90 - 1.20) | 0.97 (0.90 - 1.06) |
| Cumulative incidence at the start of the semester (approximation of prior infection) [4] | 10 additional cases per 100 persons | 0.90 (0.63 - 1.27) | 0.88 (0.7 - 1.11) | 0.82 (0.6 - 1.12) |
| Percentage of people living below 150% of the poverty line | 10 percentage points | 0.94 (0.82 - 1.07) | 1.07 (0.94 - 1.21) | 1.03 (0.85 - 1.25) |
| Percentage of people living with disability [3] | 10 percentage points | 0.89 (0.62 - 1.29) | 1.31 (0.93 - 1.87) | 0.94 (0.52 - 1.68) |
| Percentage of families with a single parent [3] | 10 percentage points | 1.23 (0.71 - 2.15) | 1.29 (0.77 - 2.17) | 1.40 (0.6 - 3.29) |
| Percentage of people identifying as an ethnic/racial minority [3] | 10 percentage points | 1.02 (0.96 - 1.08) | 0.96 (0.91 - 1.02) | 0.98 (0.9 - 1.08) |
| Percentage of people identifying as Hispanic [3] | 10 percentage points | 0.99 (0.93 - 1.05) | 1.00 (0.95 - 1.05) | 0.98 (0.90 - 1.07) |
| Percentage of people identifying as Black [3] | 10 percentage points | 1.15 (0.74 - 1.80) | 0.76 (0.49 - 1.18) | 1.09 (0.70 - 1.73) |
| Percentage of people identifying as Asian [3] | 10 percentage points | 1.04 (0.95 - 1.15) | 0.93 (0.86 - 1.02) | 1.00 (0.87 - 1.15) |
| Percentage of people living in crowded housing [3] | 10 percentage points | 0.92 (0.63 - 1.35) | 0.90 (0.65 - 1.26) | 0.96 (0.56 - 1.65) |

© 2024 Lin E et al. *JAMA Network Open.*

eReferences.


1 U.S. Census Bureau. (2022).

2 Gasparrini, A., Armstrong, B. & Kenward, M. G. Multivariate meta-analysis for non-linear and other multi-parameter associations. *Statistics in medicine* **31**, 3821-3839 (2012). https://doi.org/10.1002/sim.5471

3 Centers for Disease Control and Prevention/ Agency for Toxic Substances and Disease Registry/ Geospatial Research, Analysis, and Services Program. CDC/ATSDR Social Vulnerability Index 2018 Database California. https://www.atsdr.cdc.gov/placeandhealth/svi/data_documentation_download.html. Accessed on Feb 5, 2022.

4 USAFACTS. *Coronavirus Locations: COVID-19 Map by County and State.*, <https://usafacts.org/visualizations/coronavirus-covid-19-spread-map/> (2021).

5 CalHHS. (Sacramento, 2023).

6 Katz, J., Sanger-Katz, M. & Quealy, K. in *The New York Times* (New York, 2020).

7 Hastie, T. & Tibshirani, R. *Generalized Additive Models*. (Chapman and Hall, 1990).

8 Akaike, H. Information theory as an extension of the maximum likelihood principle–In: Second International Symposium on Information Theory (Eds) BN Petrov, F. *Csaki. BNPBF Csaki Budapest: Academiai Kiado* (1973).

9 Symonds, M. R. E. & Moussalli, A. A brief guide to model selection, multimodel inference and model averaging in behavioural ecology using Akaike's information criterion. *Behavioral Ecology and Sociobiology* **65**, 13-21 (2011). https://doi.org/10.1007/s00265-010-1037-6

10 Bilinski, A. & Hatfield, L. A. Nothing to see here? Non-inferiority approaches to parallel trends and other model assumptions. *arXiv preprint arXiv:1805.03273* (2018).

11 California Department of Public Health. *Tracking variants*, <https://www.cdph.ca.gov/Programs/CID/DCDC/Pages/COVID-19/COVID-Variants.aspx> (2021).


© 2024 Lin E et al. *JAMA Network Open.*
