# Supplementary material for: COVID-19 Incidence and Age Eligibility for Elementary School
Source: JAMA Netw Open. 2024 Nov 14;7(11):e2444836. doi: 10.1001/jamanetworkopen.2024.44836 (PMC12316186; doi:10.1001/jamanetworkopen.2024.44836)
Supplement: Supplement 2. — Data Sharing Statement [file jamanetwopen-e2444836-s002.pdf]

Data Sharing Statement

Lin. COVID-19 Incidence and Age Eligibility for Elementary School. *JAMA Netw Open*. Published November 14, 2024. doi:10.1001/jamanetworkopen.2024.44836

Data
Data available: No

Additional Information
Explanation for why data not available: Data is owned by the California Department of Public Health and may be made available via a Data Use Agreement.
